# The role of PemIK (PemK/PemI) type II TA system from *Klebsiella pneumoniae* clinical strains in lytic phage infection

**DOI:** 10.1038/s41598-022-08111-5

**Published:** 2022-03-16

**Authors:** Ines Bleriot, Lucia Blasco, Olga Pacios, Laura Fernández-García, Antón Ambroa, María López, Concha Ortiz-Cartagena, Felipe Fernández Cuenca, Jesús Oteo-Iglesias, Álvaro Pascual, Luis Martínez-Martínez, Pilar Domingo-Calap, Thomas K. Wood, María Tomás

**Affiliations:** 1 Microbiology Traslational and Multidisciplinar (MicroTM)-Research Institute Biomedical A Coruña (INIBIC), Hospital A Coruña (CHUAC), University of A Coruña (UDC), A Coruña, Spain; 2 Division of Infectious Diseases and Microbiology, University Hospital Virgen Macarena, Institute of Biomedicine of Sevilla (IBIS)/CSIC/University of Sevilla, Sevilla, Spain; 3 Reference and Research Laboratory for Antibiotic Resistance and Health Care Infections, National Centre for Microbiology, Institute of Health Carlos III, Majadahonda, Madrid, Spain; 4 Clinical Unit of Microbiology, Reina Sofía University Hospital, Department of Agricultural Chemistry, Soil Science and Microbiology, University of Cordoba, Maimónides Biomedical Research Institute of Cordoba (IMIBIC), Córdoba, Spain; 5 Instituto de Biología Integrativa de Sistemas, I2SyBio, Universitat de València-CSIC, Valencia, Spain; 6 Department of Chemical Engineering, Pennsylvania State University, University Park, PA USA; 7 Study Group on Mechanisms of Action and Resistance to Antimicrobials (GEMARA) the Behalf of the Spanish Society of Infectious Diseases and Clinical Microbiology (SEIMC), Madrid, Spain; 8 Spanish Network for Research in Infectious Diseases (REIPI) and CIBER de Enfermedades Infecciosas (CIBERINFEC), Instituto de Salud Carlos III, Madrid, Spain

**Keywords:** Developmental biology, Microbiology, Molecular biology, Molecular medicine

## Abstract

Since their discovery, toxin-antitoxin (TA) systems have captivated the attention of many scientists. Recent studies have demonstrated that TA systems play a key role in phage inhibition. The aim of the present study was to investigate the role of the PemIK (PemK/PemI) type II TA system in phage inhibition by its intrinsic expression in clinical strains of *Klebsiella pneumoniae* carrying the lncL plasmid, which harbours the carbapenemase OXA-48 and the PemK/PemI TA system. Furthermore, induced expression of the system in an IPTG-inducible plasmid in a reference strain of *K. pneumoniae* ATCC10031 was also studied. The results showed that induced expression of the whole TA system did not inhibit phage infection, whereas overexpression of the *pemK* toxin prevented early infection. To investigate the molecular mechanism involved in the PemK toxin-mediated inhibition of phage infection, assays measuring metabolic activity and viability were performed, revealing that overexpression of the PemK toxin led to dormancy of the bacteria. Thus, we demonstrate that the PemK/PemI TA system plays a role in phage infection and that the action of the free toxin induces a dormant state in the cells, resulting in inhibition of phage infections.

## Introduction

Bacteriophages, also known as “phages”, are viruses that infect bacteria. These prokaryotic viruses are considered the most abundant biological entities on Earth, occurring in all environmental niches colonized by bacteria^1,2^. Phages have traditionally been divided on the basis of their life cycle into either lytic or lysogenic phages. Most lytic phages, after infecting their host, use the bacterial machinery to replicate, transcribe and translate their nucleic acid to finally lyse the bacterial cell, releasing many viral particles. By contrast, lysogenic phages, also known as temperate phages, can integrate their genome into the bacterial chromosome or follow the lytic cycle^3^. Thus, lysogenic phages are thought to be responsible for introducing a large number of genes that provide different functions to their bacterial hosts. Accordingly, the packaging system of both types of phages has been related to horizontal gene transfer, even modulating the behaviour of the bacterial hosts, through virulence and defence genes^1^. The continuous war between phages and bacteria has led to the coevolution of both entities, so that bacteria have developed defence systems to protect themselves from the phages, while the phages in turn have developed counterstrategies to evade these systems^4^. The following are some examples of defence mechanisms that protect against continuous attacks from phages: (i) surface alterations to prevent phage adsorption, (ii) prevention of phage DNA injection, (iii) restriction of incoming DNA, (iv) acquisition of phage-specific immunity through clustered regularly interspaced short palindromic repeats (CRISPR) and (v) toxin-antitoxin (TA) systems^5^.

TA systems are widely distributed in bacteria^6,7^ and are located on bacterial chromosomes, plasmids and in phages^8^. The wide range of TA systems have been classified into eight groups (type I-VIII) based on the nature and mechanism of action of the antitoxin^9^. These systems are encoded by adjacent genes, generally consisting of two components: a stable toxin, and an unstable antitoxin, which is degraded under stress conditions by protease systems^10^, leading to toxin activation and often resulting in reduced bacterial metabolism^9^. The most prevalent kind of TA system is the type II TA system, in which both toxin and antitoxin are proteins^11^, encoded in the same operon and co-expressed^12^, and in which the antitoxin neutralizes the toxicity of the toxin by direct protein–protein interactions. Toxin targets can be very diverse, but most inhibit a central cellular process, such as translation or DNA replication^13^. Since their discovery in a plasmid in 1983 by Ogura and Sota^14^, TA systems have captivated the attention of many scientists, who have attributed to them multiple functions in cell physiology such as plasmid maintenance^14,15^, which plays a crucial role in the dissemination and evolution of antibiotic resistance, maintaining multi-resistant plasmids^8,16^. These systems can also play a role in bacterial persistence^17–20^, biofilm formation^21,22^, general stress response^23^ and phage inhibition^6,24–27^. However, today there remain many unanswered questions regarding the functions of the systems in cell physiology. Recently, we suggested that the main physiological role of TA systems is phage inhibition^9^.

However, the importance of TA systems in phage inhibition has not been widely reported. The first involvement was demonstrated for the type I TA system Hok/SoK from the R1 plasmid, which inhibited T4 phage infection, probably due to activation of the toxin after global transcription reduction by the lytic phage^24^. Additional evidence for the role of TA systems was provided years later, when the type II TA systems MazF/MazE and RnlA/RnlB were shown to inhibit infection by phage P1 and T4, respectively^25,26,28^. More recently, the discovery of the type III TA systems ToxN/ToxI in the plasmid pECA1039 of *Erwinia carotovora* revealed inhibition of phages phiA2 and phiM1^6^. Finally, the well-known abortive infection AbiEii/AbiEi system, from plasmid pNP40 and suggested to be a type IV TA system, inhibits the 936 phage family^27^.

The aim of the present research was to study the role of the type II TA system PemIK (PemK/PemI) harboured by an lncL plasmid, previously isolated and characterized by our group^29^. With this aim, the intrinsic expression of the system in clinical strains of *Klebsiella pneumoniae* carrying the OXA-48 carbapenemase was determined, together with the induced expression of the system in an IPTG-inducible expression plasmid in the reference strain of *K. pneumoniae* ATCC10031.

## Results

### Isolation, propagation and electron microscopy of phages

**Figure 1:**
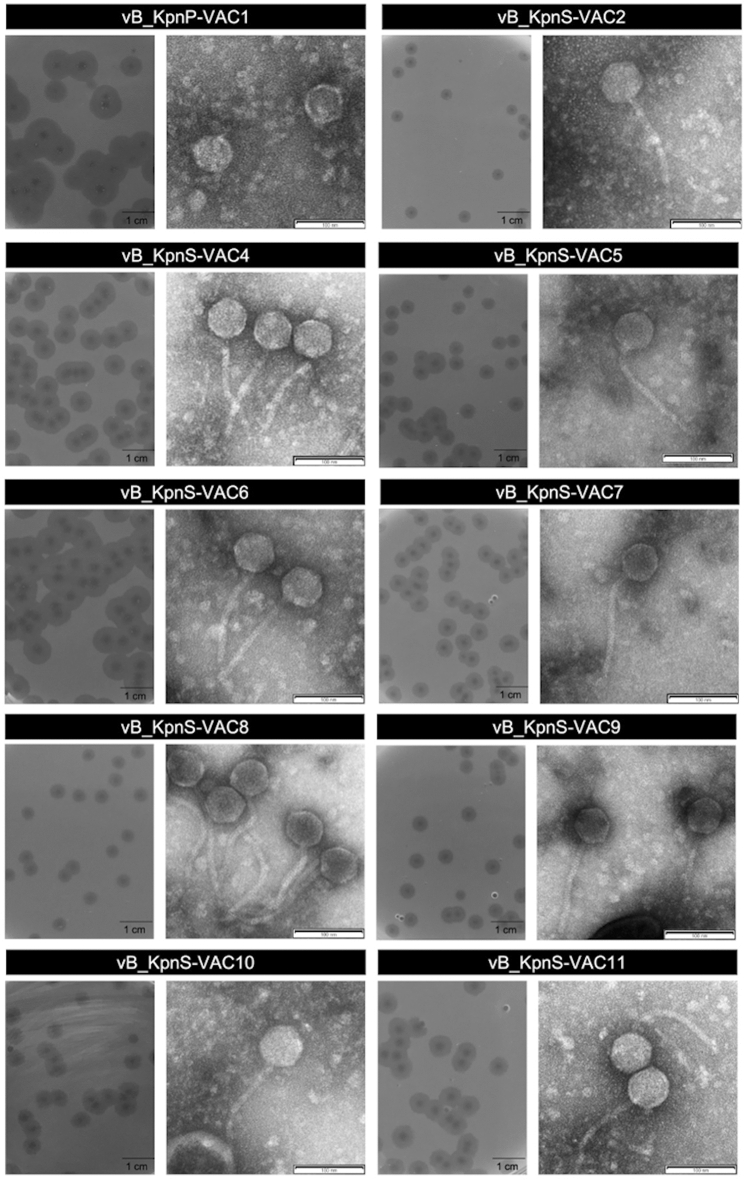
Plaque morphologies and transmission electron microscopy images showing the structure of the ten phages under study. All belong to the order *Caudovirales*. vB_KpnP-VAC1 is a member of the *Podoviridae* family, characterized by having a short tail, while all the others are members of the *Siphoviridae* family, characterized by having a large and flexible tails. The scale bar of the plaque morphologies represents 1 cm, and the scale bar of the TEM represent 100 nm.

The ten phages used in this study, named according to accepted practices^30^ (Fig. 1), were obtained from waste water samples, using as host the reference strain of *K. pneumoniae* ATCC10031, which has a capsular type KL2. Focusing on the morphology of the plaques produced by the different phages, we observed that the size of the plaques ranged from 2.5 to 7.8 mm in diameter on the lawn of ATCC10031 cells (Fig. 1). In addition, all of the plaques were surrounded by a halo that is often interpreted as an indicator of depolymerase-mediated digestion of bacterial capsules^31^. Transmission electron microscopy (TEM) images revealed that all the phages belonged to the order *Caudovirales* i.e. tailed phages of double-stranded DNA (dsDNA). All of the phages had an icosahedral head, and most had a long and flexible tail characteristic of the *Siphoviridae* family, while one phage, vB_KpnP-VAC1, had a small tail, characteristic of the *Podoviridae* family (Fig. 1).

### Phage genome sequencing

Genome sequencing revealed that all phages under study, available from the Genbank BioProject PRJNA739095 (http://www.ncbi.nlm.nih.gov/bioproject/739095) (Table 1), were lytic *Caudovirales* phages, lacking lysogenic genes such as integrases, recombinase, repressor and excisionase. In addition, sequencing corroborated the TEM results by confirming that phage vB_KpnP-VAC1 was a member of the *Podoviridae* family and that the other nine phages were members of the *Siphoviridae* family. Furthermore, sequence homology with the other phage genome sequences available in the NCBI database allowed them to be classified by genus, which revealed that phage vB_KpnP-VAC1 was a member of the genus *Teetrevirus*, while the other phages were members of the genus *Webervirus*. The phage size ranged from 39,371 to 53,113 bp, and the guanine-cytosine content ranged from 47.86% to 51.63% (Table 1). Sequence analysis revealed that the proteins were organized into functional modules within the genome, grouping them into genes related to structure, packaging, lysis, transcription and regulation. Regarding the lysis genes, all phages had endolysins and holin, both of which are proteins responsible for the degradation of the bacterial cell wall during the infection of the host; however, a difference was observed in terms of spanin (protein involved in the lysis process in gram-negative host) depending on the family to which the phage belonged, i.e. the *Siphoviridae* phages had a unimolecular spanin (U-Spanin) while the *Podoviridae* phage had a heterodimer molecule spanin (I-Spanin). Finally, regarding host capsid degradation genes, fives of the phages were found to carry depolymerase genes: two of them had two different depolymerases (vB_KpnS-VAC2 and vB_KpnS-VAC6) and three of them had a depolymerase (vB_KpnS-VAC4, vB_KpnS-VAC5 and vB_KpnS-VAC10). However, neither phage vB_KpnP-VAC1 nor phage vB_KpnS-VAC7 carried a depolymerase gene in their genome.

**Table 1:** Characteristics of the genome sequences of the ten lytic phages under study (BioProject accession number: PRJNA739095; http://www.ncbi.nlm.nih.gov/bioproject/739095).

Phages	Accession no	Family	Genus	Genome size (bp)	G + C (%)
vB_KpnP-VAC1	SAMN19773206	*Podoviridae*	*Teetrevirus*	39.371	50.75
vB_KpnS-VAC2	SAMN19773207	*Siphoviridae*	*Webervirus*	51.784	47.86
vB_KpnS-VAC4	SAMN19773215	*Siphoviridae*	*Webervirus*	45.558	51.11
vB_KpnS-VAC5	SAMN19773216	*Siphoviridae*	*Webervirus*	49.636	50.46
vB_KpnS-VAC6	SAMN19773219	*Siphoviridae*	*Webervirus*	51.554	51.63
vB_KpnS-VAC7	SAMN19773220	*Siphoviridae*	*Webervirus*	49.684	51.25
vB_KpnS-VAC8	SAMN19773224	*Siphoviridae*	*Webervirus*	48.993	50.52
vB_KpnS-VAC9	SAMN19773231	*Siphoviridae*	*Webervirus*	53.113	50.52
vB_KpnS-VAC10	SAMN19773232	*Siphoviridae*	*Webervirus*	48.935	50.65
vB_KpnS-VAC11	SAMN19773540	*Siphoviridae*	*Webervirus*	48.826	50.83

### Phage infectivity assays in solid medium

**Figure 2:**
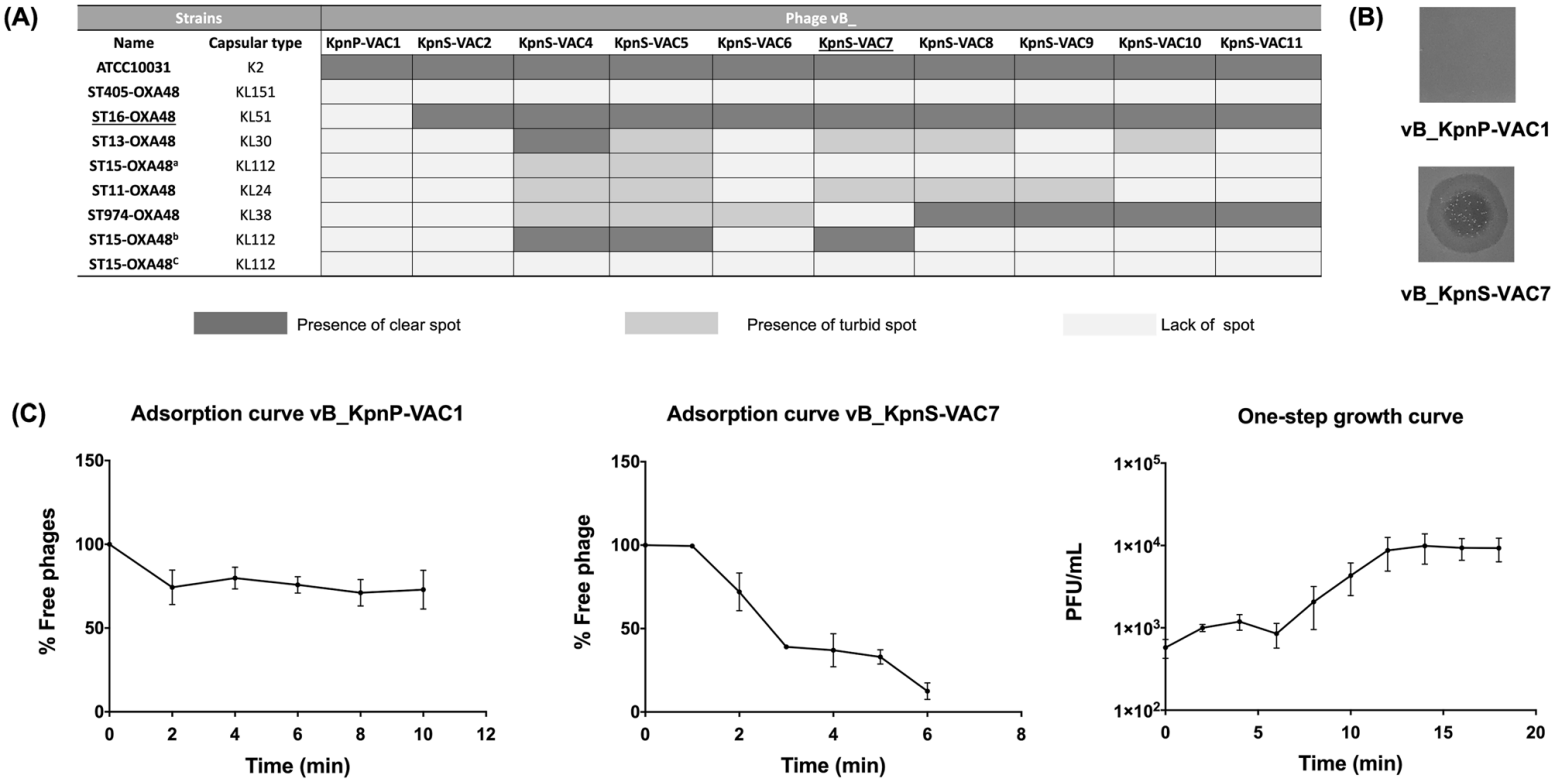
**(A)** The strain infectivity assay was performed with the ten lytic phages and the collection of clinical strains of *K. pneumoniae*, in which all strains harboured the lncL-type plasmid carrying the OXA-48 carbapenemase and the PemK/PemI TA system. The reference strain ATCC10031 was used as a control. Light grey indicates lack of spot; dark grey indicates presence of clear spot; grey indicates presence of turbid spot. **(B)** Spot test of phages vB_KpnP-VAC1 and vB_KpnS-VAC7 in the clinical strain ST16-OXA48. **(C)** Adsorption curve for phages vB_KpnP-VAC1 and vB_KpnS-VAC7, and the one-step growth curve of phage vB_KpnS-VAC7 at an MOI of 0.001 in the clinical strain ST16-OXA48. *L* latent time; *B* burst-size.

The phage infectivity assay in solid medium was performed using the spot test technique. The clinical strains of *K. pneumoniae* in the collection were thus tested to determine their susceptibility to phages relative to the susceptibility of the reference strain ATCC10031 (Fig. 2A). All clinical strains in the collection harbour the lncL-type plasmid carrying the carbapenemase OXA-48 and the PemK/PemI TA system, and they have different capsular types. The results obtained showed that the reference strain ATCC10031 (capsular type KL2) was susceptible to all phages, while in the clinical strains, 61.25% of the infectious events produced no infection, 17.5% of the infectious events produced weak infection and 21.25% of the infectious events produced effective infection. Accordingly, clinical strain ST16-OXA48, which has a capsular type KL51, was the strain most susceptible to phages, as it was infected by 9 different phages. By contrast, the clinical strains ST405-OXA48 (capsular type KL151) and ST15-OXA48^c^ (capsular type KL112) were not infected by any of the phages assayed. On the basis of these results, we selected the susceptible clinical strain ST16-OXA48 and two lytic phages according to their infectivity capacity for the remaining experiments: vB_KpnP-VAC1 (no infectivity) and vB_KpnS-VAC7 (high infectivity) (Fig. 2B).

### Intrinsic study of the role of the PemK/PemI type II TA system on the clinical strains of *K. pneumoniae*

#### Phage adsorption and one-step growth curve

First, phage adsorption to the bacterial surface receptors was studied with the previously selected strain and phages. Phage vB_KpnP-VAC1 showed slight adsorption, with 27.08% of phage adsorbed at 10 min on strain ST16-OXA48, while phage vB_KpnS-VAC7 showed a high percentage of adsorption, with 88.69% phage adsorbed after 6 min (Fig. 2C). Accordingly, a one-step growth curve was constructed for phage vB_KpnS-VAC7 to determine the latent period (i.e. time taken for a phage particle to reproduce inside an infected host cell) and the burst size (i.e. number of viral particles released in each infection cycle per cell), which were respectively 6 min and 15 ± 2 PFU (Fig. 2C). In the case of phage vB_KpnP-VAC1, a one-step growth curve was not constructed, because the phage was not able to successfully infect clinical strain ST16-OXA48.

#### Infectivity assay in liquid medium: infection curve

**Figure 3:**
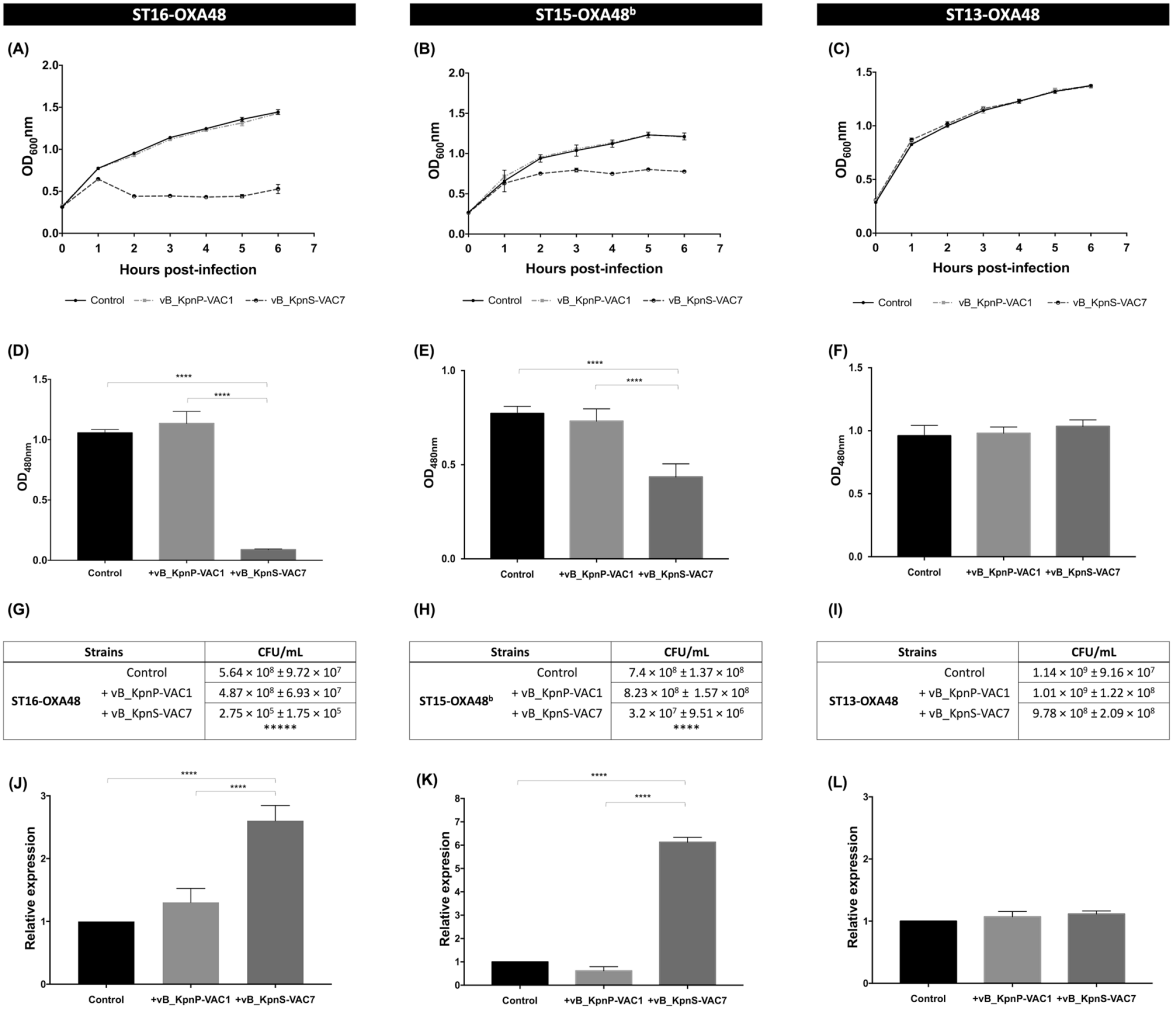
(**A–C**) Infection curves for the strains ST16-OXA48, ST15-OXA48^b^ and ST13-OXA48 with phages vB_KpnP-VAC1 and vB_KpnS-VAC7 at an MOI of 0.1. **(D**–**F)**. Measurement of metabolic activity of strains ST16-OXA48, ST15-OXA48^b^ and ST13-OXA48 by the colorimetric assay (WST-1 based) after 2 h of infection with phages vB_KpnP-VAC1 and vB_KpnS-VAC7 at an MOI of 0.1. **(G**–**I)** Measurement of viability by CFU/mL counts of strains ST16-OXA48, ST15-OXA48^b^ and ST13-OXA48 after 2 h of infection with phage vB_KpnP-VAC1 and vB_KpnS-VAC7. **(J**–**L)** Measurement of the relative expression of the toxin *pemK* with respect to the antitoxin *pemI* in the clinical strains ST16-OXA48, ST15-OXA48^b^ and ST13-OXA48 by qRT-PCR after 30 min of phage infection with vB_KpnP-VAC1 and vB_KpnS-VAC7 at an MOI of 0.1. In all cases, the strain without phage infection was the control, and the errors bars represent the standard deviation of the three experimental replicates. In both the metabolic activity, viability and relative expression assays, strains ST16-OXA48 and ST15-OXA48^b^ show significant differences between the strains infected with phage vB_KpnS-VAC7 and the other two strains (****p-value < 0.001), while no significant differences were observed between the control and the strain infected with phage vB_KpnP-VAC1 (p-value > 0.05). However, in the case of the ST13-OXA48 strain, no significant differences were observed between the different strains (p-value > 0.05).

The infectivity assay in liquid medium was tested by constructing infection curves for phages vB_KpnP-VAC1 and vB_KpnS-VAC7 at the multiplicity of infection (MOI) of 0.1 in the selected clinical strain of *K. pneumoniae* ST16-OXA48 (Fig. 3A). The results of the phage infection curve analysis confirmed those obtained in the infectivity assay in solid medium: the growth of the strain ST16-OXA48 was inhibited when infected with the phage vB_KpnS-VAC7 but not with the phage vB_KpnS-VAC1, when its growth was similar to that of the control strain without infection. In order to confirm that this effect also occurred in other clinical strains, an infection curve was constructed for clinical strain ST15-OXA48^b^, which displayed the same pattern of susceptibility to phages vB_KpnP-VAC1 and vB_KpnS-VAC7 in solid medium as strain ST16-OXA48. The infection curve for this strain showed the same growth inhibition phenomenon in the presence of the phage vB_KpnS-VAC7 (Fig. 3B). We then wanted to find whether this phenomenon also occurred in those strains in which susceptibility to the phage vB_KpnS-VAC7 was lower (turbid spot in the solid medium infectivity assay). We selected the ST13-OXA48 strain for this purpose. The infection curve showed that in liquid medium the phage vB_KpnS-VAC7 was not able to infect the strain and thus neither inhibit bacterial growth (Fig. 3C).

#### Enzymatic assay using the cell proliferation reagent WST-1 and measurement of viability

The assay, which measures the ubiquitous reducing agents NADH and NADPH as biochemical markers to assess the metabolic activity of the cell, revealed that strains ST16-OXA48 and ST15-OXA48^b^ not infected by phage and infected with phage vB_KpnP-VAC1 showed a metabolic activity reaching a OD_480nm_ = 1.057 ± 0.026 and 1.136 ± 0.098 for strain ST16-OXA48 (Fig. 3D) and 0.772 ± 0.038 and 0.732 ± 0.065 for the strain ST15-OXA48^b^ (Fig. 3E) as well as a high number of CFU/mL (Fig. 3G,H) after 2 h of infection. By contrast, strains ST16-OXA48 and ST15-OXA48^b^ infected with phage vB_KpnS-VAC7 showed a significant reduction in metabolic activity (OD_480nm_ > 0.01 and OD_480nm_ < 0.5, respectively), resulting in a significantly lower number of CFU/mL (Fig. 3G,H). In addition, the infection curves showed growth inhibition. Therefore, these data seem to indicate that infection with the phage vB_KpnS-VAC7 leads to a metabolic inactive state of the bacterial cells of the ST16-OXA48 and ST15-OXA48^b^ strains. On the other hand, in the case of strain ST13-OXA48 no significant differences were observed in terms of metabolic activity (0.950 ± 0.082, 0.980 ± 0.049 and 1.034 ± 0.051) (Fig. 3F) or CFU/mL counts (Fig. 3I) with and without phage infection, as neither of the two phages produced effective infection in this strain.

#### Relative expression of the PemK/PemI TA system by qRT-PCR

The study of the relative expression of the *pemK* toxin with respect to the *pemI* antitoxin revealed different expression patterns depending on whether the strain was successfully or unsuccessfully infected by the phages (Fig. 3J–L). When phage vB_KpnS-VAC7 was able to produce a successful infection in strains ST16-OXA48 and ST15-OXA48^b^ a significant overexpression of *pemK* toxin was observed with respect to *pemI* antitoxin (2.60-fold and 6.13-fold, respectively). However, when the phage was not able to produce a successful infection, as in the case of phage vB_KpnP-VAC1 in strains ST16-OXA48, ST15-OXA48^b^ and ST13-OXA48 or phage vB_KpnS-VAC7 in strain ST13-OXA48, no significant difference in toxin expression with respect to antitoxin was observed (value close to 1).

#### Bioinformatic analysis of the PemK and PemI protein

To further study the PemK and PemI proteins in the clinical strain ST16-OXA48 after infection with phages vB_KpnP-VAC1 and vB_KpnS-VAC7, a bioinformatic analysis of the proteins was carried out. We used the ProtParam tool (https://web.expasy.org/cgi-bin/protparam/protparam) to determine the molecular weight, the isoelectric point (pI) and the instability index of both proteins. This study revealed that the PemI antitoxin was composed of a total of 85 amino acids with an estimated molecular weight of 9.24853 kDa and a pI of 4.69, while PemK was composed of a total of 110 amino acids with a molecular weight of 11.82368 kDa and a pI of 9.98. Regarding the stability of the proteins, the PemI antitoxin has an instability index of 48.93, while the PemK toxin has an instability index of 30.33. According to the cut-off value determined by ProtParam, the antitoxin is classified as unstable, with an instability index higher than 40, while the toxin is classified as stable, with an instability index lower than 40. In view of these data, both qualitative and quantitative assays of the proteins were performed in the clinical strain ST16-OXA48 infected with phages vB_KpnP-VAC1 and vB_KpnS-VAC7.

#### LC–MS and NanoUHPLC-Tims-QTOF analysis

Qualitative results obtained by LC–MS of the clinical strain ST16-OXA48 without phage infection and infected with the phages vB_KpnP-VAC1 and vB_KpnS-VAC7 revealed the presence of proteins in the range of 4–20 kDa. This size is within the range in which PemK and PemI proteins should be present, according to the theoretical weight determined *in silico*. In accordance with these results, a sample of ST16-OXA48 strains without phage infection and infected with phages vB_KpnP-VAC1 and vB_KpnS-VAC7 were quantitatively analysed by NanoUHPLC-Tims-QTOF. The results showed that in the case of infection with phage vB_KpnS-VAC7, the PemK toxin (Avg. Mass: 11.824 kDa) was present with an area value of 9.83 × 10^2^, while in the presence of phage vB_KpnP-VAC1 and in the control, the toxin was not detected. Presence of the antitoxin was not detected in any case, probably due to its high instability. Focusing on the general study of the protein profile of strain ST16-OXA48 infected with phage vB_KpnS-VAC7 relative to the uninfected strain and to the infected strain with phage vB_KpnP-VAC1 (Table 2 and Table S1, Supplementary table), in the case of infection with phage vB_KpnS-VAC7, the strain had a greater amount of proteins of the plasmid lncL. On the other hand, the assays confirmed that in presence of this phage, the cells were in a state of cellular stress, due to the successful phage infection, presenting a greater quantity and presence of stress proteins, such as abortive phage infection protein (area value: 1. 22 × 10^4^ *vs* 4.02 × 10^2^ in the case of the control strain and 2.42 × 10^2^ in the case of the strain infected with phage vB_KpnP-VAC1), phage shock protein (area value: 2.58 × 10^3^ while in the other cases it is not detected) and oxidative stress proteins. This stress state was also reflected by the greater amount and presence of *Quorum sensing* proteins, such as autoinducer 2 aldose (area value: 3.99 × 10^4^) or autoinducer-2 (AI-2) (area value: 4.91 × 10^3^), which were not detected in the other cases. Regarding transcription and replication, greater amounts of protein were observed, highlighting the presence of the DNA starvation/stationary phase protection protein Dps (area value: 1.00 × 10^5^ *vs* 1.38 × 10^3^ in the case of the control and 9.96 × 10^2^ in the case of the strain infected with phage vB_KpnP-VAC1). At the same time, the metabolism, specifically the Krebs cycle, appeared very active, with a great abundance of associated proteins, with the values always being higher in the strain infected with the phage vB_KpnS-VAC7 than in the others. The high abundance of transport-related proteins, nucleotide metabolism (both catabolism and synthesis), and protein/amino acid degradation should also be noted. Moreover, the cell wall/membrane was strongly activated, which may suggest that virions had taken control of the bacterial cells to form new viral particles. Finally, a large abundance of proteins related to cell cycling and cell division was observed, but was not detected in the other cases.

**Table 2:** Quantitative data on the protein profile of the clinical strain of *K. pneumoniae* ST16-OXA48 not infected and infected with the phages: vB_KpnP-VAC1 and vB_KpnS-VAC7.

Description	Accession	− 10lgP	Area ST16-OXA48			Avg. Mass
			Control	vB_KpnP-VAC1	vB_KpnS-VAC7	
**lncL plasmid**						
Beta-lactamase OXA-48 partial [*Klebsiella pneumoniae* subsp. *pneumoniae*]	AIS93798.1	16,389	0.00E+00	ND	1.82E+09	28,063
Recombinase (plasmid) [*Klebsiella pneumoniae* subsp. *pneumoniae*]	AZJ02352.1	7108	ND	ND	4.02E+06	35,529
PemK toxin [*Klebsiella pneumoniae* subsp. *pneumoniae*]	OWU94844.1	52.08	ND	ND	9.82E+02	11,824
**Defense mechanism**						
Abortive phage infection protein [*Klebsiella pneumoniae* subsp. *pneumoniae*]	OXU78716.1	145.44	4.02E+02	2.42E+02	1.22E+04	50,868
Phage shock protein [*Klebsiella pneumoniae* subsp. *pneumoniae*]	AIW76278.1	99.65	2.58E+02	0.00E+00	2.58E+03	25,444
Autoinducer 2 aldolase Quorum sensing [*Klebsiella pneumoniae* subsp. *pneumoniae*]	AMA26955.1	155.35	0.00E+00	ND	3.99E+04	32,384
Autoinducer-2 (AI-2) modifying protein LsrG Quorum sensing [*Klebsiella pneumoniae* subsp. *pneumoniae*]	AMV55201.1	82.38	ND	ND	4.91E+03	11,517
**Oxidative stress**						
Heat shock protein 90 [*Klebsiella pneumoniae* subsp. *pneumoniae*]	AIW69779.1	217.79	9.46E+02	4.02E+02	7.72E+04	71,093
Oxidative damage protection protein [*Klebsiella pneumoniae* subsp. *pneumoniae*]	OCO07259.1	126.51	3.22E+03	2.69E+02	1.07E+04	10,919
**Transcription and replication**						
DNA starvation/stationary phase protection protein Dps [*Klebsiella pneumoniae* subsp. *pneumoniae*]	OAK82820.1	196.08	1.38E+03	9.96E+02	1.00E+05	18,708
DNA gyrase subunit B [*Klebsiella pneumoniae* subsp. *pneumoniae*]	AMA17435.1	145.76	ND	2.44E+02	1.42E+04	90,087
**Metabolism**						
Pyruvate dehydrogenase (acetyl-transferring) homodimeric type [*Klebsiella pneumoniae* subsp. *pneumoniae*]	OCN38258.1	190.51	ND	ND	8.48E+04	99,434
Succinate dehydrogenase flavoprotein subunit [*Klebsiella pneumoniae* subsp. *pneumoniae*]	AIK80411.1	192	ND	ND	4.27E+04	64,478
**Nucleotide metabolism**						
Uridine phosphorylase [*Klebsiella pneumoniae* subsp. *pneumoniae*]	KHF63849.1	114.75	ND	ND	1.18E+04	27,053
Exodeoxyribonuclease III [*Klebsiella pneumoniae* subsp. *pneumoniae*]	OCN92642.1	78.33	ND	ND	1.08E+03	30,898
**Protein degradation and amino acid**						
Glycine–tRNA ligase subunit beta [*Klebsiella pneumoniae* subsp. *pneumoniae*]	AMA32722.1	167.82	ND	ND	2.29E+04	76,331
Clp protease ClpX [*Klebsiella pneumoniae* subsp. *pneumoniae*]	AIW75269.1	146.82	7.39E+02	4.12E+02	1.45E+04	46,294

*Description* The protein header information as seen in the NCBI database (https://www.ncbi.nlm.nih.gov). *Accession* The accession number of the protein as seen in the NCBI database, *-10LgP* the protein confidence score. *Area* the area under the curve of the peptide feature found at the same *m/z* and retention time as the MS/MS scan. This can be used as an indicator of the abundance and *Avg. Mass* is the protein mass calculated using the average mass.

### Study of the role of the PemK/PemI type II TA system in the transformed strains of *K. pneumoniae* ATCC10031 by induced expression

#### Induced expression of TA system

**Figure 4:**
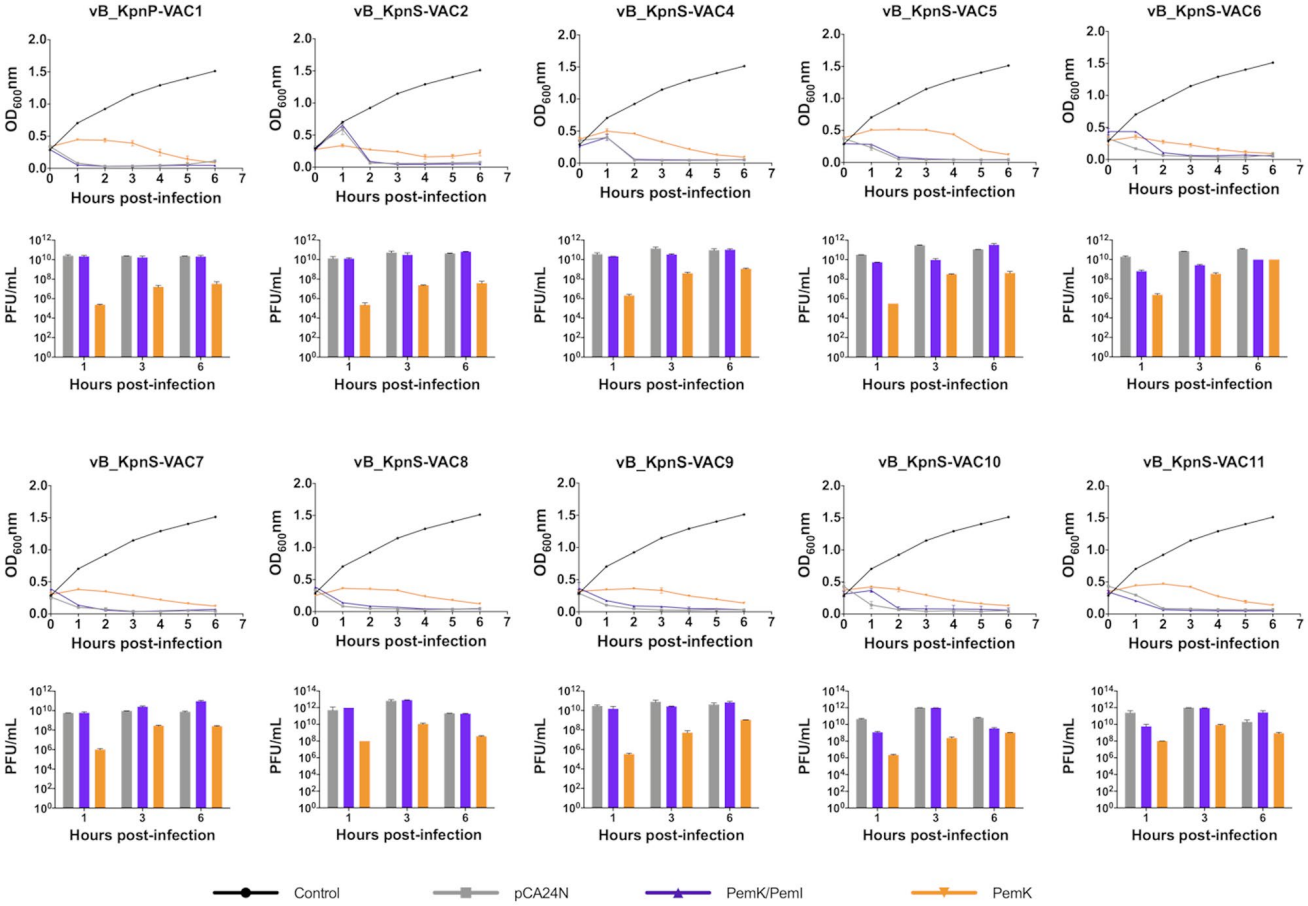
Infection curve for transformed strain ATCC10031/pCA24N, ATCC10031/pCA24N (*pemK/pemI*) and ATCC10031/pCA24N (*pemK*) in the presence of the ten lytic phages, and enumeration of PFU/mL after 1, 3 and 6 h of infection. The strain ATCC10031/pCA24N, harbouring the empty plasmid without phage infection was used as a control. In all cases there was a significant difference (p-value < 0.05) between the PFU/mL of the strain ATCC10031/pCA24N (*pemK*) and the strains ATCC10031/pCA24N and ATCC10031/pCA24N (*pemK/pemI*). The errors bars represent the standard deviation of the three experimental replicates.

In order to further investigate the role that the *pemK/pemI* system can play in phage infection, induced expression assays were performed with the transformed strains ATCC10031/pCA24N, ATCC10031/pCA24N (*pemK/pemI*) and ATCC10031/pCA24N (*pemK*) in the presence of ten lytic phages at an MOI of 0.1 (Fig. 4). As shown in all the curves, overexpression of the whole *pemK/pemI* TA locus did not inhibit any of the phages, and there was no significant difference compared to the strain with the empty plasmid. However, overexpression of the *pemK* toxin alone inhibited infection by all ten phages during the first hours. These results were corroborated by the significantly lower PFU/mL values after 1 h post-infection for strain ATCC10031/pCA24N (*pemK*) than in the strains ATCC10031/pCA24N and ATCC10031/pCA24N (*pemK/pemI*) (Fig. 4). Furthermore, a significant increase in PFU/mL of the ATCC10031/pCA24N (*pemK*) strain was observed at 3 h post-infection, which corresponds to the observed decrease in OD_600nm_, and thus to the phage infection of the strain, suggesting a reversible effect of the toxin in arresting the viral life cycle.

**Figure 5:**
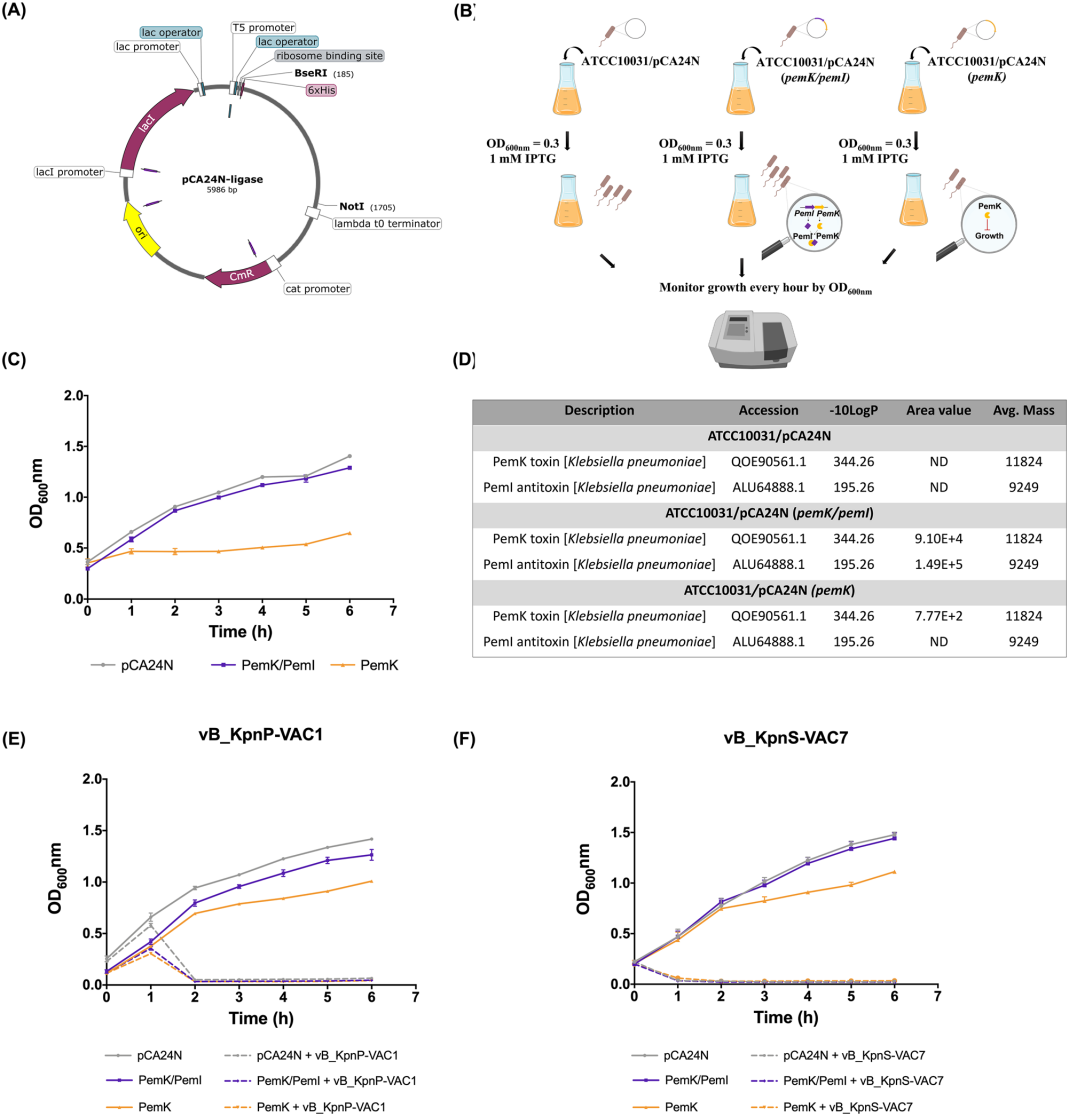
**(A)** Graphical representation of the pCA24N plasmid, made with Snapgene software, showing the location of the restriction enzymes used to analyse the transformed strains ATCC10031/pCA24N (*pemK/pemI*) and ATCC10031/pCA24N (*pemK*). **(B)** Schematic overview of growth curves. **(C)** Growth curves of the strains ATCC10031/pCA24N, ATCC10031/pCA24N (*pemK/pemI*) and ATCC10031/pCA24N (*pemK*) in the presence of 1 mM IPTG added at early logarithmic phase. The strain ATCC10031/pCA24N, harboring the empty-plasmid, was used as a control. The errors bars represent the standard deviation of the three experimental replicates. **(D)** Quantitative data of expression of the PemK toxin and PemI antitoxin in the transformed strains ATCC10031/pCA24N, ATCC10031/pCA24N (*pemK/pemI*) and ATCC10031/pCA24N (*pemK*) after 3 h of induction with 1 mM of IPTG. *Description* The protein header information as seen in the NCBI database (https://www.ncbi.nlm.nih.gov). *Accession* The accession number of the protein as seen in the NCBI database, − *10LgP* the protein confidence score. *Area* the area under the curve of the peptide feature found at the same *m/z* and retention time as the MS/MS scan. This can be used as an indicator of the abundance and *Avg. Mass* is the protein mass calculated using the average mass. (**E**,**F**) Infection curves of ATCC10031/pCA24N, ATCC10031/pCA24N (*pemK/pemI*) and ATCC10031/pCA24N (*pemK*) strains without IPTG induction in the presence of phage vB_KpnP-VAC1 **(E)** and vB_KpnS-VAC7 **(F)** at an MOI of 0.1. Strains without phage infection and without IPTG induction were used as controls to see the kinetics of each strain. Error bars represent the standard deviation of the three experimental replicates.

To rule out that the effect observed in the different infection curves was only due to IPTG induction, induction controls without infection (Fig. 5A–C), as well as an infection control with phages vB_KpnP-VAC1 and vB_KpnS-VAC7 without IPTG induction (Fig. 5E,F) were included. Overexpression of *pemK* in the strain ATCC10031/pCA24N (*pemK*) inhibited bacterial growth during the first three hours until the strain regrew to twice the OD_600nm_ at 6 h after induction, while overexpression of the whole *pemK/pemI* TA system in the strain ATCC10031/pCA24N (*pemK/pemI*) led to normal growth, with no significant differences compared to the strain ATCC10031/pCA24N (Fig. 5C). For the non-induced control, in the absence of overexpression, the phage inhibited bacterial growth in all the strains: ATCC10031/pCA24N, ATCC10031/pCA24N (*pemK/pemI*) and ATCC10031 (*pemK*) (Fig. 5E,F).

#### Protein analysis of TA system overexpression over time by sodium dodecyl sulphate–polyacrylamide gel electrophoresis (SDS-PAGE)

Induced overexpression of the *pemK/pemI* TA system and *pemK* toxin over time was also studied by protein analysis on a 15% SDS-PAGE gel in the transformed strains ATCC10031/pCA24N, ATCC10031/pCA24N (*pemK/pemI*) and ATCC10031/pCA24N (*pemK*) (Fig. S1, Supplementary Figure). The analysis confirmed the overexpression of the *pemK/pemI* system with a band at 10–15 kDa after 1, 3 and 6 h of induction with IPTG. In addition, the level of overexpression was highest after 3 h, resulting in a more intense band. However, in the case of overexpression of the *pemK* toxin, protein expression was not observed under comparable experimental conditions, due to growth inhibition by the toxin, the same phenomenon observed in the study of Bukowski et al. (2013)^32^. This effect was more pronounced after 3 h. However, after 6 h, a slight increase in the amount of protein was observed due to the slight regrowth of the strain.

#### LC–MS and NanoUHPLC-Tims-QTOF analysis

In order to quantify the production of PemK toxin and PemI antitoxin in the transformed strains ATCC10031/pCA24N (*pemK/pemI*) and ATCC10031/pCA24N (*pemK*) after 3 h of induction with IPTG, a NanoUHPLC-Tims-QTOF was performed, after having detected the presence of proteins in the range of 4–20 kDa by LC–MS. The results showed that the strain ATCC10031/pCA24N (*pemK/pemI*) showed the presence of PemK (Avg. Mass: 11.824 kDa) and PemI (Avg. Mass: 9.249 kDa) with an area value of 9.10 × 10^4^ and 1.49 × 10^5^ respectively, while in the case of the strain ATCC10031/pCA24N (*pemK*) only the PemK toxin was present, as expected, with an area value of 7.7 × 10^2^ (Fig. 5D). In the control strain ATCC10031/pCA24N none of these proteins were present.

#### Enzymatic assay using the cell proliferation reagent WST-1 and measurement of viability

**Figure 6:**
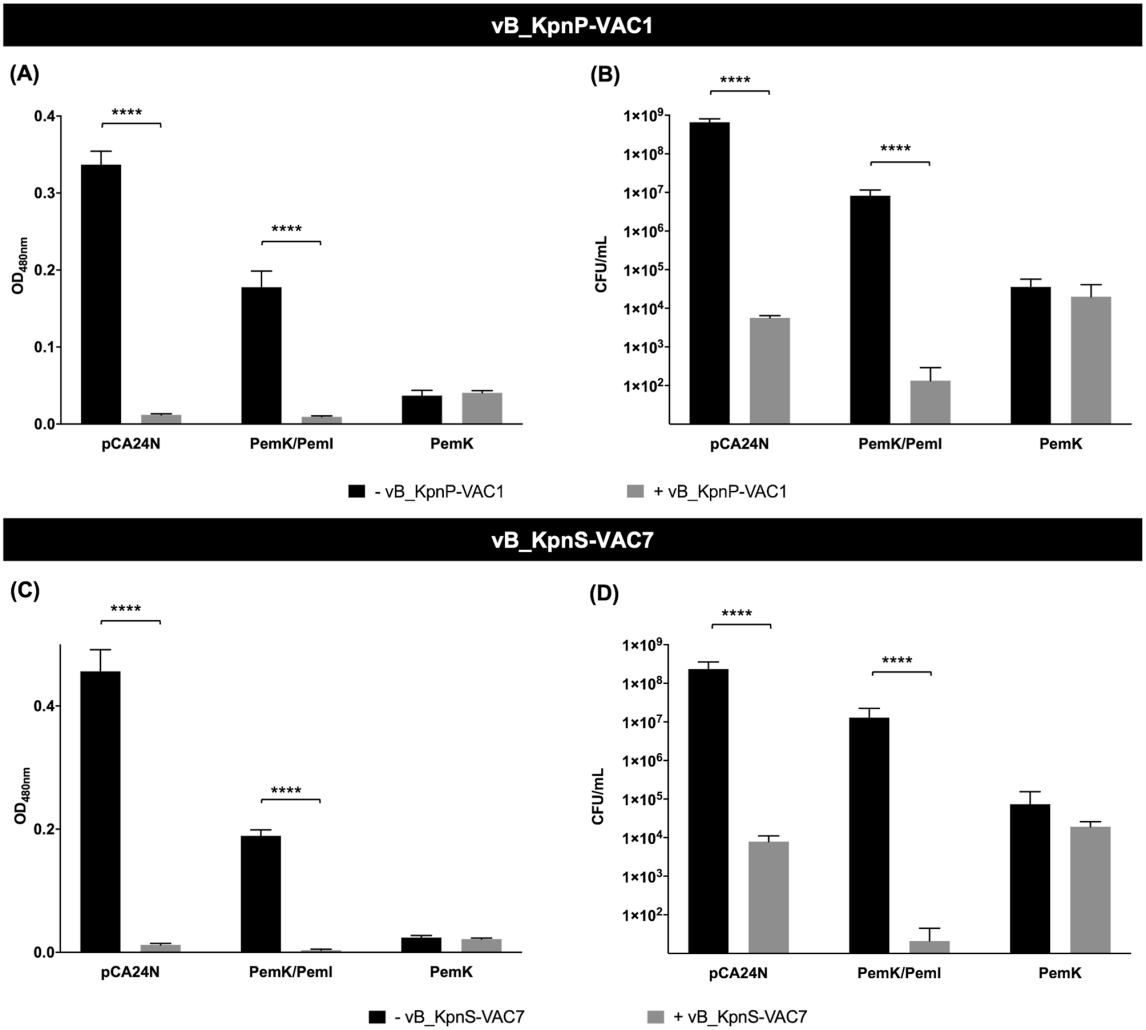
(**A**,**C**) Measurement of the metabolic activity by the colorimetric assay (WST-1 based) and (**B**,**D**) measurement of viability by CFU/mL counts of the transformed strains ATCC10031/pCA24N, ATCC10031/pCA24N (*pemK/pemI*) and ATCC10031/pCA24N (*pemK*) after induction with 1 mM IPTG for 2 h and infection with phages vB_KpnP-VAC1 (**A**,**B**) and vB_KpnS-VAC7 (**C**,**D**) at an MOI of 0.1. The controls were the transformed strain without phage infection. In all cases, no significant differences (p-value > 0.05) were observed between the strain ATCC10031/pCA24N (*pemK)* infected with phages and the uninfected strain, in terms of metabolic activity and cell viability, possibly indicating that the cells are in a state of cell dormancy. ****p-value < 0.001. The errors bars represent the standard deviation of the three experiment replicates.

The assay that measures the metabolic activity revealed that the transformed strains ATCC10031/pCA24N as well as ATCC10031/pCA24N (*pemK/pemI*) without phage infection showed a metabolic activity reaching a OD_480nm_ = 0.337 ± 0.018 and 0.178 ± 0.021, respectively, in the case of the experiment with phage vB_KpnP-VAC1 (Fig. 6A) and OD_480nm_ = 0.456 ± 0.035 and 0.189 ± 0.097 in the case of experiment with phage vB_KpnS-VAC7 (Fig. 6C), after induction with IPTG for 2 h. These data were corroborated by the high numbers of CFU/mL, 6.60 × 10^8^ ± 1.47 × 10^8^ and 8.22 × 10^6^ ± 3.39 × 10^6^ CFU/mL in the case of infection with phage vB_KpnP-VAC1 (Fig. 6B) and 2.33 × 10^8^ ± 1.23 × 10^8^ and 1.29 × 10^7^ ± 9.57 × 10^6^ CFU/mL in the case of infection with phage vB_KpnS-VAC7 (Fig. 6D). By contrast, as expected, the strains ATCC10031/pCA24N and ATCC10031/pCA24N (*pemK/pemI*) infected with phages vB_KpnP-VAC1 (Fig. 6A) and vB_KpnS-VAC7 (Fig. 6C) lacked metabolic activity (OD_480nm_ < 0.01), indicating cell death due to phage infection, as reflected in the sharp decrease in CFU/mL: 5.67 × 10^3^ ± 8.00 × 10^2^ and 2.40 × 10^2^ ± 1.33 × 10^2^ CFU/mL in the case of infection with phage vB_KpnP-VAC1, and 7.90 × 10^3^ ± 3.27 × 10^3^ and 2.11 × 10^1^ ± 2.42 × 10^1^ CFU/mL in the case of infection with phage vB_KpnS-VAC7. However, for strain ATCC10031/pCA24N (*pemK*), we observed that the strain lacked metabolic activity both with and without phage infection (OD_480nm_ > 0.01). Furthermore, the number of CFU/mL was not significantly different in cultures with and without phage infection: 3.60 × 10^4^ ± 2.08 × 10^4^ and 2.00 × 10^4^ ± 2.10 × 10^4^ CFU/mL in the case of infection with phage vB_KpnP-VAC1 and 7.42 × 10^4^ ± 8.15 × 10^4^ and 1.92 × 10^4^ ± 6.90 × 10^3^ CFU/mL in the case of infection with phage vB_KpnS-VAC7. Thus, this data indicates that overexpression of the toxin leads to a dormant state of the cell, which prevents phage infection.

## Discussion

Recent studies have revealed that the key role of TA systems in bacterial cell physiology may be related to phage inhibition. In this regard, here we demonstrate the involvement of the type II TA system PemK/PemI in the reversible inhibition of phage infection caused by cell dormancy due to the effect of the free toxin.

First, focusing on the phages studied, TEM and sequencing revealed that they were all lytic phages belonging to the order *Caudovirales*, i.e. dsDNA-tailed bacteriophages, most being members of the genus *Webervirus* belonging to the family *Siphoviridae* and one member of the genus *Teetrevirus* belonging to the family *Podoviridae*.

Regarding the intrinsic study, the phage infectivity assay in solid medium revealed that a high percentage of infective events resulted in no infection (61.25%) or only weak infection (17.5%) in the clinical strains, relative to infection in the reference strain *K. pneumoniae* ATCC10031. Therefore, given that all clinical strains in this study harbour the PemK/PemI TA system, we reasoned that the type II TA system may be involved in the inhibition of phage infection. The intrinsic study focused on two lytic phages, which-despite being able to bind to the bacterial surface receptors of clinical strain ST16-OXA48, as demonstrated by adsorption curves showed different patterns of infectivity in the solid medium and liquid infectivity assay: phage vB_KpnP-VAC1 did not show any successful infection, whereas phage vB_KpnS-VAC7 produced successful infection with inhibition of bacterial growth. Notably, similar phenomena in which adsorption to bacterial surface receptors does not explicitly imply successful infection have already been described in the literature^33^. Next, the relative expression of the *pemK* toxin with respect to the *pemI* antitoxin by qRT-PCR revealed a correlation between the infection of the strain by the phage and the overexpression of the toxin. Indeed, when the phage is able to produce a successful infection of the strain, as is the case of vB_KpnS-VAC7 with strains ST16-OXA48 and ST15-OXA48^b^, the toxin was overexpressed. In the same line, the quantitative study of protein profile by NanoUHPLC-Tims-QTOF of strain ST16-OXA48 without phage infection and infected with phages vB_KpnP-VAC1 and vB_KpnS-VAC7 revealed the presence of the toxin PemK only in the case of infection with phage vB_KpnS-VAC7. In addition, the presence of the PemI antitoxin was not detected in any case, which may be due to its high instability. However, the fact that PemK toxin only appears in the strain where phage is able to successfully establish infection seems to indicate that this TA system may be involved in the inhibition of phage infection as a defence mechanism, even though successful infection of the strain occurred in this case. On the other hand, the general study of the protein profile of strain ST16-OXA48 infected with phage vB_KpnS-VAC7 compared to the uninfected strain and to the strain in presence of phage vB_KpnP-VAC1 revealed a greater amount of proteins encoded by the plasmid lncL, which harbours the carbapenemase OXA-48 and the PemK/PemI system. This seems to indicate that the strain activates its plasmids as a defence mechanism against phage infection. Furthermore, the study findings also revealed the high level of cellular stress that the bacterial cells are undergoing, due to the successful infection of the strain by the phage vB_KpnS-VAC7, producing greater amounts of stress proteins such as oxidative stress proteins or the phage shock protein, already well described in the literature as a stress protein that is activated by phage infection in *E. coli* strains^34^. In addition, *Quorum sensing* proteins, previously described as protecting against phage infection^35^, were also present. On the other hand, focusing on the general processes of bacterial cells, we observed greater amounts of transcription and replication proteins. We believe that the differences we observed in transcription and replication between the ST16-OXA48 strain infected with phage vB_KpnS-VAC7 and the other strains were mainly due to the growth phase of the cells. In the case of the strains ST16-OXA48 without phage infection and infected with phage vB_KpnP-VAC1, as they were in the stationary phase, transcription and replication were somewhat reduced^36^. However, in the case of strain ST16-OXA48 infected with phage vB_KpnS-VAC7, growth was inhibited due to phage infection, as observed in the infection curve, together with its metabolic activity as observed in the metabolic assay. Furthermore, it showed low levels of transcription and replication, which we hypothesized could be due to some vestigial uninfected cells, or to the phage taking control of the bacterial replication machinery for the generation of its new virions. One of the proteins present in the strain infected with phage vB_KpnS-VAC7 is related to DNA starvation, which may be associated with the entry into dormancy and the lack of metabolic activity observed previously. Finally, we observed a large number of proteins that we believe are related to the elaboration of new virions, such as proteins involved in the metabolism of nucleotides, proteins or amino acids, as well as a strong presence of transporters.

To better understand the role of the PemK toxin in phage inhibition, we studied the induced expression of the TA system using an IPTG-inducible expression plasmid instead of performing a *pemK/pemI* knockout. Indeed, a knockout was not feasible in this case because the TA system is carried by a plasmid, which makes it very difficult to eliminate all copies of the system. Therefore, the study revealed that overexpression of the *pemK* toxin led to inhibition of early stage phage infection. By contrast, overexpression of the complete *pemK/pemI* TA system, with the PemK toxin blocked by the antitoxin PemI*,* did not confer any protection against phage infection (similar to the empty plasmid control, as well as the transformed strain without IPTG induction). Toxins of type II TA systems have been widely shown to cause down-regulation of cell metabolism as gene transcription is inhibited^20,29^. In this case, the metabolic measurement by the enzymatic assay with the WST-1 reagent as well as the enumeration of CFU/mL in the viability assay revealed that overexpression of *pemK* toxin led to the dormancy of bacterial cells after 2 h of induction with IPTG. However, bacteriophages require the machinery of their bacterial hosts for replication^38^. Therefore, as the cell is in a dormant state, the bacteriophage cannot replicate and is unable to proliferate. This was observed by enumeration of the PFU/mL of the ATCC10031/pCA24N (*pemK*) strain, in which the phage counts were significantly lower than in the strains ATCC10031/pCA24N and ATCC10031/pCA24N (*pemK/pemI*). These results confirm that the presence of free toxin^37,39^ protects the bacteria against phage infection during the first hours by inducing dormancy of the bacterial cells. At the same time, in all the infection curves of the ATCC10031/pCA24N (*pemK*) strain, we observed a reversible effect of viral cycle arrest, similar to that shown by Moreno del-Aramo et *al.* 2020^40^. Indeed, after 3 h post-infection the phages were able to infect the strain, resulting in a decrease in the OD_600nm_ (opposite effect to that observed in the presence of IPTG induction and absence of phage infection) and an increase in phage counts (PFU/mL). This could be explained by possible autoregulation of the toxin through its endoribonuclease activity^41,42^, as previously described in the homologous system MazF/MazE type II TA system, in which MazF toxin cleaves its own mRNA in the presence of stress, thus autoregulating its expression^43^. Alternatively, it could be due to the fact that maximum overexpression occurs at 3 h post-induction with IPTG, as demonstrated by protein analysis by SDS-Page, and that the amount of toxin then decreases, leading to infection of the strain. The bacterium would then return to a metabolically active stage, allowing infection by the phages remaining in the medium.

## Conclusion

This is the first study concerning the role of the type II TA system PemK/PemI harboured by the lncL-type plasmid in phage infection. The study findings demonstrate that the dormancy of bacterial cells due to the action of *pemK* toxin leads to reversible inhibition of phage infection. This captivating field is still in its infancy and further research is thus required for a better understanding of the relationship between TA systems and phage infection.

## Materials and methods

### Bacterial strains and plasmid

A collection of clinical strains of *K. pneumoniae* carrying the lncL-type plasmid harbouring the OXA-48 carbapenemase and the PemK/PemI TA system was used to study the intrinsic role of this TA system in phage infection (Table 3). The capsular type of each strain was determined by consulting the Kaptive website (https://kaptive-web.erc.monash.edu). In addition, the reference strain *K. pneumoniae* subsp*. pneumoniae* (ATCC10031) obtained from the American Culture Collection was used in this study, as well as the transformed strains ATCC10031/pCA24N, ATCC10031/pCA24N (*pemK/pemI*) and ATCC10031/pCA24N (*pemK*) (Table 3), to study the induced expression of the PemK/PemI type II TA system during phage infection. The ATCC10031 strains were transformed with the pCA24N (Cm^R^, Lacl^q^) expression plasmid^44^ (inducible by IPTG and constructed by our group) harbouring the complete *pemK/pemI* TA system and the *pemK* toxin alone^29^. All of the strains were grown in Luria–Bertani (LB) medium, and in the case of the transformed strains the medium was supplemented with chloramphenicol (30 μg/mL) (LB-CM) to maintain the plasmid.

**Table 3:** Bacterial strains and plasmids used in this study.

Strain or plasmid	Main characteristics	Source or reference
ATCC10031	*K. pneumoniae subsp. pneumoniae* reference strain	ATCC
ATCC10031/pCA24N	*K. pneumoniae subsp. pneumoniae* reference strain with expression plasmid pCA24N	This study
ATCC10031/ pCA24N (*pemK/pemI*)	*K. pneumoniae subsp. pneumoniae* reference strain with expression plasmid pCA24N with the type II TA system *pemK/pemI*	This study
ATCC10031/ pCA24N (*pemK*)	*K. pneumoniae* reference strain with expression plasmid pCA24N with the toxin *pemK*	This study
pCA24N	Expression plasmid cm^R^, LacIq	^44^
pCA24N (*pemK/pemI*)	Expression plasmid pCA24N with the TA system *pemK/pemI*	^29^
pCA24N (*pemK*)	Expression plasmid pCA24N with the TA system *pemK*	^29^
ST405-OXA48	Clinical strain isolated from wound (Genbank accession no. WRXJ00000000) harbouring the carbapenemase OXA-48 and the TA system *pemK/pemI*	^1^
ST16-OXA48	Clinical strain isolated from Urine (Genbank accession no. WRXF00000000) harbouring the carbapenemase OXA-48 and the TA system *pemK/pemI*	^1^
ST13-OXA48	Clinical strain isolated from rectal sample (Genbank accession no. WRWZ00000000) harbouring the carbapenemase OXA-48 and the TA system *pemK/pemI*	^1^
ST15-OXA48^a^	Clinical strain isolated from axillary smear (Genbank accession no. WRWX00000000) harboring the carbapenemase OXA-48 and the TA system *pemK/pemI*	^1^
ST11-OXA48	Clinical strain isolated from urine (Genbank accession no. WRWW00000000) harbouring the carbapenemase OXA-48 and the TA system *pemK/pemI*	^1^
ST974-OXA48	Clinical strain isolated from urine (Genbank accession no. WRWT00000000) harbouring the carbapenemase OXA-48 and the TA system *pemK/pemI*	^1^
ST15-OXA48^b^	Clinical strain isolated from blood harbouring the carbapenemase OXA-48 and the TA system *pemK/pemI*	This study
ST15-OXA48^c^	Clinical strain isolated from blood harbouring the carpabemase OXA-48 and the TA system *pemK/pemI*	This study

### Isolation and propagation of the lytic phages

Ten lytic phages isolated from environmental water samples were used in this study. Briefly, water samples (50 mL) were taken near sewage plants and kept at room temperature until processing in the laboratory. Once in the laboratory, the samples were vortexed and centrifuged 4000 × g 10 min. The supernatant was recovered and filtered through with 0.45 μm and 0.22 μm filters, to remove the cells and debris. An aliquot (1 mL) of the filtered samples was then added to 500 μL of *K. pneumoniae* ATCC10031 in 4 mL soft agar (0.5% NaCl, 1% tryptone and 0.4% agar supplemented with 1 mM CaCl2) and poured onto TA agar plates (0.5% NaCl, 1% tryptone and 1.5% agar; supplemented with 1 mM CaCl_2_), in the double-layer method. Plates were incubated at 37 °C. Isolated plaques of different morphology (i.e. plaque size and presence of a surrounding halo) were then recovered by picking with a micropipette and stored at − 70 °C. In order to check and purify the isolated plaques, two additional plaque assays and plaque picking steps were performed.

### Propagation of phage and transmission electron microscopy

Plaque-purified phages were amplified in LB liquid media supplemented with 1 mM CaCl_2_ (LB-CaCl_2_), with shaking (180 rpm) at 37 °C, by infecting an early logarithmic growth phase culture of ATCC10031 (OD_600nm_ = 0.3–0.4). After lysis, i.e. when the culture appeared clear, bacteria and debris were removed by centrifugation (4302 × g 10 min) and filtration (0.45 μm). Finally, the supernatants were titrated by the double-layer method by serial dilution in SM buffer (0.1 M NaCl, 10 mM MgSO_4_, 20 mM Tris–HCL pH 7.5) and stored at 4 °C. Part of each solution of high titre phages was negatively stained with 1% aqueous uranyl acetate and then analyzed by transmission electron microscopy in a JEOL JEM-1011 electron microscope.

### Phage DNA extraction and whole genome sequencing (WGS)

Genomic phage DNA was isolated from the strains with the phenol:chloroform method following the phage hunting protocol (https://phagesdb.org/media/workflow/protocols/pdfs/PCI_SDS_DNA_Extraction_2.2013.pdf). DNA concentrations and quality were measured in a Nanodrop ND-10000 spectrophotometer (NanoDrop Technologies, Waltham, MA, USA) and Qubit fluorometer (Thermo Fisher Scientific, USA). Genomic libraries were then prepared using the Nextera XT Library prep kit (Illumina), following the manufacturer’s instructions, and the distribution of fragments lengths was checked in the Agilent 2100 Bioanalyser using the Agilent Hight sensitivity DNA kit. Libraries were purified using the Mag-Bind RXNPure plus magnetic beads (Omega Biotek) and finally, the pool was sequenced in Miseq platform (Illumina Inc, USA). The quality of the FASTQ files was checked using the software FastQC^45^ and summarized using MultiQC^46^. Sequences of 300 bp paired-end reads of each isolate were assembled *“*de novo*”* with Spades V.3.15.2^47^. All assembly were annotated by sequence homology using Patric 3.6.9 (http://www.patricbrc.org), Blastx (http://blast.ncbi.nlm.nih.gov) and HHmer (http://hmmer.org). In addition, the HHpred tool (https://toolkit.tuebingen.mpg.de/tools/hhpred), which predicts functions through protein structure, was also used. The family and genus of the different phages were determined by sequence homology with the phage sequences available in the NCBI database. Complete genome sequences were included in GenBank BioProject PRJNA739095 (http://www.ncbi.nlm.nih.giv/bioproject/739095).

### Phage infectivity assay in solid medium

A phage infectivity assay in solid medium was carried out by the spot test technique^48^, in the collection of clinical strain of *K. pneumoniae* carrying the lncL-type plasmid harbouring the carbapenemase OXA-48 and the *pemK/pemI* TA system*.* The reference strain ATCC10031, which lacks both the carbapenemase OXA-48 and the *pemK/pemI* TA system, was used as a control. Briefly, 200 μL of an overnight culture was mixed with 4 mL soft agar and poured onto TA agar plates. Once the soft medium had solidified, 15 μL drops of high titre phages were added to the plates. For each strain, a negative control consisting of SM buffer was included in each plate. All determinations were performed in triplicate. The following criteria were used to determine the phage infectivity: lack of spot, presence of clear spot and presence of turbid spot.

### Phage adsorption to bacterial host cells ST16-OXA48

Adsorption of vB_KpnP-VAC1 and vB_KpnS-VAC7 phage to the bacterial surface receptors of clinical strain ST16-OXA48 was determined from the adsorption curve^49^. Briefly, an overnight culture of *K. pneumoniae* ST16-OXA48 was diluted 1:100 in LB-CaCl_2_, and incubated at 37 °C at 180 rpm, until a cell count of 10^8^ CFU/mL was reached. At this point, the cultures were left at room temperature in the absence of agitation and infected with a phage suspension at an MOI of 0.001. Every minute, 1 mL of culture was collected and placed in contact with 1% chloroform. Subsequently, the samples were centrifuged for 2 min at 12,000 × g to sediment the cell debris and adsorbed phage. Serial dilutions of the supernatant were made in SM buffer for subsequent plating on a double agar plate with the corresponding host plating strain. In the case of phage vB_KpnP-VAC1, the plating host was natural host of this phage, the reference strain ATCC10031, because it was not able to produce successful infection in clinical strain ST16-OXA48. However, in the case of vB_KpnS-VAC7 the plating host was clinical strain ST16-OXA48. The number of phages mixed with bacterial host cells at time 0 was considered 100% free of phages. The adsorption curve analysis was performed in triplicate.

### One-step growth curve assay of vB_KpnS-VAC7 in the clinical strain ST16-OXA48

A one-step growth curve of phage vB_KpnS-VAC7 was constructed for the clinical strain ST16-OXA48, in order to determine the latent period and the burst size. The latent period was defined as the interval between adsorption of the phages to the bacterial cells and the release of phage progeny. The burst size of the phage was defined as number of viral particles released in each cycle of infection per bacterial cells. For this purpose, an overnight culture of *K. pneumoniae* ST16-OXA48 was diluted 1:100 in LB-CaCl_2_ and incubated at 37ºC at 180 rpm, until reaching a cell count of 10^8^ CFU/mL. At this point, the culture was infected with a phage suspension at an MOI of 0.001 and left at room temperature for 4 min (adsorption time). At the end of the adsorption time, the culture was washed by two successive 10 min centrifugations at 6000 × g in order to remove the free phages. After washing, the pellet was resuspended with LB-CaCl2 and 25 μl of bacterial mixture was added to 25 mL of LB-CaCl2 (time 0) and incubated at 37 °C with shaking. Aliquots (1 mL) of the culture were collected every 2 min for 18 min, and serial dilutions were made in SM buffer and subsequently seeded on double agar plates for subsequent quantification. The one-step growth curve analysis was performed in triplicate.

### Phage infectivity assay in liquid medium and overexpression of TA system and infection with phage collection

Infection curves were constructed from the selected clinical strain of *K. pneumoniae* ST16-OXA48, as well as for the clinical strain ST15-OXA48^b^, ST13-OXA48 and the transformed strains ATCC10031/pCA24N, ATCC10031/pCA24N (*pemK/pemI*) and ATCC10031/pCA24N (*pemK)* to study the effect of the TA system on phage infection, considering both intrinsic and induced expression of the TA system. For this purpose, an overnight culture of the strains was diluted 1:100 in LB-CaCl_2_ or LB-CM-CaCl_2_, respectively and incubated at 37 °C at 180 rpm, until reaching the early logarithmic phase. At this point, the culture was induced by 1 mM of IPTG, in the case of the transformed strains, and infected with phages at an MOI of 0.1, in both cases. The only difference was that the clinicals strain were only infected with two phages, which were selected according to the results of the strain infectivity assay in solid medium: vB_KpnP-VAC1, which did not produce a spot, and vB_KpnS-VAC7, which produced a clear spot in the clinical strain ST16-OXA48. The optical density was measured every hour for 6 h, and the number of PFU/mL was determined at 1, 3 and 6 h after phage infection by the double-layer method, in the case of the transformed strains. The strains not infected with phage were used as controls. Moreover, for the transformed strain, a control without IPTG-mediated induction was also included to enable determination of the kinetics of each strain. These strains were also included with phages vB_KpnP-VAC1 and vB_KpnS-VAC7 without induction in order to determine the kinetics of infection under these conditions. All analyses were performed in triplicate. The data were statistically analyzed using the Graphpad (Prism 8) software by two-way ANOVA followed by multiple comparisons by post hoc Tukey’s test at a significance level of P > 95%.

### Enzymatic assay using the cell proliferation Reagent WST-1

The metabolic activity of the clinical strains ST16-OXA48, ST15-OXA48^b^ and ST13-OXA48 and the transformed strains ATCC10031/pCA24N, ATCC10031/pCA24N (*pemK/pemI*) and ATCC10031/pCA24N (*pemK*) after 2 h of infection with phages vB_KpnP-VAC1 and vB_KpnS-VAC7 was analysed using a colorimetric enzymatic assay based on the water-soluble tetrazolium salt (WST-1) reagent and electron mediators (Roche, Mannheim, Germany). Tetrazolium salts are widely used in cell biology for measuring the metabolic activity of various type of cells, ranging from mammalian to microbial origin^50,51^. Briefly, the cultures were incubated at 37 °C at 180 rpm, until the culture reached an early logarithmic phase. At this point, the culture was incubated with 1 mM of IPTG, in the case of the transformed strains, and infected with phages at an MOI of 0.1, in both cases. After 2 h of infection and two washes, the culture cells (OD_600nm_ = 0.1) were placed in a 96-well polystyrene plate (Corning Incorporated, NY, USA) and 10 μL/well of the reagent was added. After incubation for 1 h at 37 °C without shaking and shaking (180 rpm) for 10 min, the optical density was measured at OD_480nm_. In all cases controls were the strains without phage infection. The OD_480nm_ of the medium culture (LB) in the presence of WST-1 reagent was used to normalize all data. All experiments were performed in triplicate. For the statistical analysis, the multiple *t*-test (P > 95%) was used with the Graphpad (Prism 8) software.

### Measurement of viability

Viability was measured in the clinical strains ST16-OXA48, ST15-OXA48^b^ and ST13-OXA48 and in the transformed strains ATCC10031/pCA24N, ATCC10031/pCA24N (*pemK/pemI*) and ATCC10031/pCA24N (*pemK*) after 2 h of infection with phages vB_KpnP-VAC1 and vB_KpnS-VAC7 by counting the CFU/mL. Briefly, cultures of the strains were incubated at 37 °C at 180 rpm, until the culture reached an early logarithmic phase. At this point, the culture was incubated with 1 mM IPTG, in the case of the transformed strains, and infected with phages at an MOI of 0.1, in both cases. The number of CFU/mL was determined after 2 h of infection. In all cases, the controls were the strains without phage infection. All experiments were performed in triplicate. For statistical analysis a multiple *t*-test (P > 95%) was implemented in Graphpad software (Prism 8).

### RNA extraction of the selected clinical isolates

RNA extraction was performed using the high purity RNA isolation kit (Roche, Mannheim, Germany) from samples taken from the infection curves of clinical strains ST16-OXA48, ST15-OXA48^b^ and ST13-OXA48 after 30 min of phage infection with vB_KpnP-VAC1 and vB_KpnS-VAC7 at an MOI of 0.1. The resulting extract was subsequently quantified in a NanoDrop ND-10000 spectrophotometer (NanoDrop Technologies, Waltham, MA, USA) and the concentration was adjusted to 50 ng/μL. All experiments were performed in triplicate.

### Measurement of relative expression of the TA system by qRT-PCR in the selected clinical isolates

The determination of the relative expression of the *pemK* toxin with respect to the antitoxin *pemI* after 30 min of phages infections at an MOI of 0.1 in the clinical strains ST16-OXA48, ST15-OXA48^b^ and ST13-OXA48, was carried out by qRT-PCR with a Lightcycler 480 RNA MasterHydrolisis Probe (Roche, Mannheim, Germany), under the following conditions: reverse transcription at 63 °C for 3 min, denaturation at 95 °C for 30 s, followed by 45 cycles of 15 s at 95 °C and 45 s at 60 °C and, finally, cooling at 40 °C for 30 s. The UPL primers and probes used for the analysis are shown in Table 4. For statistical analysis of the data, a 1.5-fold cut-off value was applied to identify differentially expressed TA genes. For each strain, the expression of all genes, was normalized relative to the housekeeping gene, *recA* and each experiment was performed for triplicate^52^.

**Table 4:** Primers and probes used in this study.

Primer Name	Sequences	Probes	References
***pemI* (Antitoxin)**			
pemI_Fow	CAGACGCCCGCAGTATTC	102/	This study
pemI_Rev	GCCGAGATTTCAGCGTTC	102/	This study
***pemK* (Toxin)**			
pemK_Fow	CCGGACGATCGATATGAAAG	142/	This study
pemK_Rev	GTCAGGATGGTGGCCAGA	142/	This study
***recA* (Housekeeping gene)**			
recA_Fow	GCCGAATTCCAGATCCTCTA	148/	This study
recA_Rev	TCTTTCACGCCGAGGTCTAC	148/	This study

### Bioinformatic analysis of the protein PemK and PemI

The physical and chemical characteristics of PemK toxin and PemI antitoxin were determined by bioinformatics analysis conducted using the ProtParam tool (https://web.expasy.org/protparam/) for subsequent proteomics experiments. This tool enables calculation of molecular weight, theoretical pI, amino acid composition, atomic composition, extinction coefficient, estimated half-life, instability index, aliphatic index and GRAVY.

### LC–MS and NanoUHPLC-Tims-QTOF analysis

LC–MS and NanoUHPLC-Tims-QTOF analyses were performed for qualitative and quantitative study of the protein profile of strain ST16-OXA48 without and with phage infection with vB_KpnP-VAC1 and vB_KpnS-VAC7, as well as to study the protein expression of PemK/PemI type II TA system and the PemK toxin alone in the transformed strains ATCC10031/pCA24N, ATCC10031/pCA24N (*pemK/pemI*) and ATCC10031/pCA24N (*pemK*) after induction with 1 mM IPTG for 3 h. First of all, the samples were prepared for that purpose an overnight culture of strains was diluted 1:100 in 25 mL LB, and incubated at 37 °C at 180 rpm, until the cultures reached an early logarithmic phase. The cultures of clinical strain ST16-OXA48 were then infected with phages at an MOI of 0.1. Meanwhile, cultures of the transformed strains were incubated with 1 mM of IPTG. After 3 h of infection/induction, the cultures were harvested and centrifuged at 4302×*g* for 20 min at 4 °C. The pellets were then stored at − 80 °C to facilitate cell disruption. The next day, the pellet was resuspended in PBS and sonicated. Finally, the sonicated pellets were centrifuged at 4302×*g* for 20 min at 4 °C, and the flow-through, now called crude extract, was used for LC–MS and NanoUHPLC-Tims-QTOF analyses. The equipment used for this purpose was a TimsTof Pro mass spectrophotometer (Bruker), a nanoESI source (CaptiveSpray), a time-QTOF analyser and a nanoELUTE chromatograph (Bruker). Sample preparation was carried out by tryptic digestion in solution with reduction-alkylation followed by Ziptip desalting. Data were aquired in nanoESI positive ionisation mode, Scan PASEF-MSMS mode, CID fragmentation mode, with an acquisition range of 100–1700 m/z. the products were separated on a Reprosil C18 column (150 × 0.075 mm, 1.9 µm and 120 Å) (Bruker) at 50 °C, with an injection volume of 2 µL. The mobile phases consisted of 0.1% H_2_O/formic acid (A) and 0.1% acetonitrile/formic acid (B). The flow rate was 0.4 µL/min, and the gradient programme was as follows: 11% B (0–5 min), 16% B (5–10 min), 35% B (10–16 min), 95% B (16–18 min) and 95% B (18–20 min). Finally, different software was used for data acquisition: Compass HyStar 5.1 (Bruker) and TimsControl (Bruker), DataAnalysis (Bruker) and PEAKS studio (Bioinformatics Solutions).

### Protein analysis of TA system overexpression over time by sodium dodecyl sulphate–polyacrylamide gel electrophoresis (SDS-PAGE)

Overexpression of the PemK/PemI TA system and PemK toxin over time was studied by protein analysis on a 15% SDS-PAGE gel. For this purpose, an overnight culture of the transformed strains ATCC10031/pCA24N, ATCC10031/pCA24N (*pemK/pemI*) and ATCC10031/pCA24N (*pemK*) was diluted 1:100 in 75 mL LB and incubated at 37 °C at 180 rpm until the cultures reached early logarithmic phase, when they were then incubated with 1 mM IPTG. After induction for 1, 3 and 6 h, 25 mL of culture was harvested and centrifuged at 4302×*g* for 20 min at 4 °C. The pellets were stored at − 80 °C. The next day, the pellets were resuspended in PBS and the cells were ruptured by sonication. The sonicated pellets were centrifuged at 4302×*g* for 20 min at 4 °C. The resulting crude extracts were quantified in a NanoDrop ND-10000 spectrophotometer (NanoDrop Technologies, Waltham, MA, USA). Finally, crude extract was loaded onto a 15% SDS-page gel for protein separation. The resulting gel was stained with Coomasie Brillant Blue R-250 overnight and finally the gel was destained by successive washing with 40% methanol. The control was the crude extract of the strain ATCC10031/pCA24N.

## Supplementary Information

Supplementary Figure S1.

Supplementary Table S1.
