# Supplementary material for: The role of PemIK (PemK/PemI) type II TA system from Klebsiella pneumoniae clinical strains in lytic phage infection
Source: Sci Rep. 2022 Mar 16;12:4488. doi: 10.1038/s41598-022-08111-5 (PMC8927121; doi:10.1038/s41598-022-08111-5)
Supplement: Supplementary file 1 — Supplementary Figure S1. [file 41598_2022_8111_MOESM1_ESM.pdf]

Supplementary Figure.

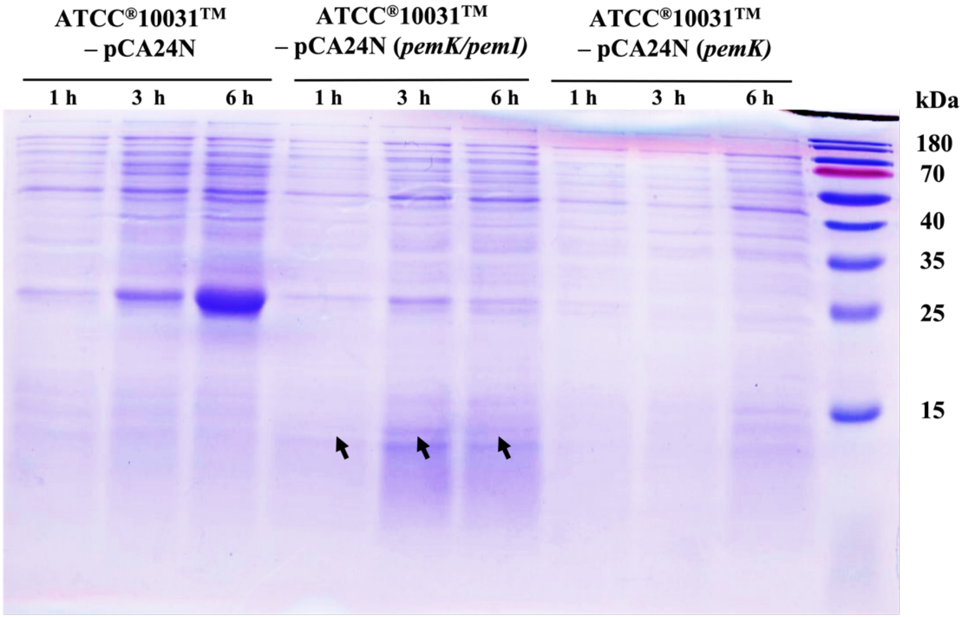

Figure S1. Protein analysis of the TA system over time after 1, 3 and 6 h of induction with 1 mM IPTG using a 15 % SDS-PAGE gel. The bands corresponding to the PemK toxin are marked with an arrow in strain ATCC®10031™/pCA24N (*pemK/pemI*). In the case of strain ATCC®10031™/pCA24N (*pemK*) no PemK toxin is observed due to growth inhibition mediated by overexpression of the toxin. In both cases, it is observed that at 3 h of induction with IPTG, the maximum over-expression of the PemK/PemI system and of the toxin alone occurs, resulting in the ATCC®10031™/pCA24N (*pemK/pemI*) strain in a band with a higher intensity and in the ATCC®10031™/pCA24N (*pemK*) strain in a lower protein expression due to the maximum growth inhibition. The protein profile of strain ATCC®10031™/pCA24N was taken as a control.
