# Supplementary material for: The role of PemIK (PemK/PemI) type II TA system from Klebsiella pneumoniae clinical strains in lytic phage infection
Source: Sci Rep. 2022 Mar 16;12:4488. doi: 10.1038/s41598-022-08111-5 (PMC8927121; doi:10.1038/s41598-022-08111-5)
Supplement: Supplementary file 2 — Supplementary Table S1. [file 41598_2022_8111_MOESM2_ESM.pdf]

Supplementary table.

Table S1. Complete quantitative data on the protein profile of the clinical strain of *K. pneumoniae* ST16-OXA48 without and with phage infection with the vB_KpnP-VAC1 and vB_KpnS-VAC7 phages. **Description:** the protein's header information as seen in the NCBI database (https://www.ncbi.nlm.nih.gov), **Accession:** the accession number of the protein as seen in the NCBI database, **-10LgP:** the protein confidence score, **Coverage (%)**: the percentage of the protein sequence that is covered by supporting peptides, **Area:** the area under the curve of the peptide feature found at the same *m/z* and retention times as the MS/MS scan. This can be used as indicator of the abundance, **#peptide:** the number of high-confidence supporting peptides, **#Seq:** the total number of spectra identified that support the given protein and **Avg. Mass:** the protein mass calculated using the average mass.

Description	Accession	-10lgP	Coverage (%)	Coverage (%)			Area			#Peptides	#Spec			Avg. Mass
				Control	vB_KpnP-VAC1	vB_KpnS-VAC7	Control	vB_KpnP-VAC1	vB_KpnS-VAC7		Control	vB_KpnP-VAC1.1	vB_KpnS-VAC7.2	
**IncL plasmid**														
beta-lactamase OXA-48 partial [*Klebsiella pneumoniae* subsp. *pneumoniae*]	AIS93798.1	163.89	41	4	0	38	0.00E+00	ND	1.82E+05	17	1	0	44	28063
histidine phosphatase super family protein [*Klebsiella pneumoniae* subsp. *pneumoniae*]	AIK80071.1	153.7	33	0	0	25	ND	Nd	3.38E+04	11	0	0	17	45504
DUF1496 domain-containing protein [*Klebsiella pneumoniae* subsp. *pneumoniae*]	RUL14892.1	173.46	30	12	19	27	6.22E+01	0.00E+00	7.57E+03	8	1	1	10	13685
recombinase (plasmid) [*Klebsiella pneumoniae* subsp. *pneumoniae*]	AZJ02352.1	71.08	17	0	0	14	ND	ND	4.02E+03	5	0	0	4	35529
MobC (plasmid) [*Klebsiella pneumoniae* subsp. *pneumoniae*]	AZJ02355.1	152.26	61	32	32	51	1.45E+03	1.10E+03	4.51E+03	8	4	5	5	13623
DsbC family protein [*Klebsiella pneumoniae* subsp. *pneumoniae*]	RNX82348.1	58.18	7	3	3	7	0	0	4.46E+03	2	1	1	2	34664
plasmid stability family protein [*Klebsiella pneumoniae* subsp. *pneumoniae*]	ROB78068.1	96.93	35	29	18	18	3.00E+03	2.01E+03	3.28E+03	4	4	2	4	16159
DotD/TraH family lipoprotein (plasmid) [*Klebsiella pneumoniae* subsp. *pneumoniae*]	QFP85447.1	75.07	15	8	8	8	2.76E+02	0	2.57E+03	3	1	1	1	18665
MULTISPECIES: conjugal transfer protein TraW [Enterobacterales]	WP_004187486.1	102.89	18	0	3	15	ND	0	1.92E+03	6	0	1	5	43266
phospholipase D family protein (plasmid) [*Klebsiella pneumoniae* subsp. *pneumoniae]*	QWC36073.1	78.08	15	0	0	15	ND	ND	1.20E+03	2	0	0	2	20028
conjugal transfer protein [*Klebsiella pneumoniae* subsp. *pneumoniae*]	OWU94875.1	69.11	14	0	0	10	ND	ND	5.31E+02	3	0	0	2	29205
conjugal transfer protein [*Klebsiella pneumoniae* subsp. *pneumoniae*]	ROC15632.1	45.92	5	0	0	5	ND	ND	6.65E+02	1	0	0	1	29456
sugar ABC transporter substrate-binding protein (plasmid) [*Klebsiella pneumoniae* subsp. *pneumoniae*]	PJR18410.1	57.93	31	0	0	16	ND	ND	7.93E+02	2	0	0	1	9786
mRNA interferase PemK [*Klebsiella pneumoniae* subsp. *pneumoniae*]	OWU94844.1	52.08	25	0	0	25	ND	ND	9.82E+02	2	0	0	2	11824
TraO [*Klebsiella pneumoniae* subsp. *pneumoniae*]	RUL14877.1	67.55	6	0	0	6	ND	ND	4.72E+02	2	0	0	3	47512
**Defense mechanism**														
abortive phage infection protein [*Klebsiella pneumoniae* subsp. *pneumoniae*]	OXU78716.1	145.44	36	3	3	31	4.02E+02	2.42E+02	1.22E+04	17	1	1	20	50868
phage shock protein [*Klebsiella pneumoniae* subsp. *pneumoniae*]	AIW76278.1	99.65	20	6	6	13	2.58E+02	0	2.58E+03	5	2	2	3	25444
type I restriction-modification system subunit M [*Klebsiella pneumoniae* subsp. *pneumoniae*]	OYF54248.1	89.34	14	0	0	9	ND	ND	1.60E+03	6	0	0	4	57138
type I restriction-modification system subunit M [*Klebsiella pneumoniae* subsp. *pneumoniae*]	THM44413.1	71.11	5	0	0	5	ND	ND	9.24E+02	2	0	0	2	60437
autoinducer 2 aldolase Quorum sensing [*Klebsiella pneumoniae* subsp. *pneumoniae*]	AMA26955.1	155.35	49	5	0	48	0	ND	3.99E+04	14	1	0	20	32384
autoinducer-2 (AI-2) modifying protein LsrG Quorum sensing [*Klebsiella pneumoniae* subsp. *pneumoniae*]	AMV55201.1	82.38	43	0	0	33	ND	ND	4.91E+03	4	0	0	5	11517

tRNA (cytosine(32)/uridine(32)-2'-O)-methyltransferase TrmJ [*Klebsiella pneumoniae* subsp. *pneumoniae*]	OCO37558.1	58.07	16	0	0	16	ND	ND	2.02E+03	3	0	0	3	26828
(dimethylallyl)adenosine tRNA methylthiotransferase [*Klebsiella pneumoniae* subsp. *pneumoniae*]	AIW75644.1	53.92	7	0	0	4	ND	ND	7.62E+02	3	0	0	3	53551
tRNA (uracil-5-)-methyltransferase [*Klebsiella pneumoniae* subsp. *pneumoniae*]	AIX82212.1	47.67	4	0	0	4	ND	ND	5.25E+02	1	0	0	2	41859
tRNA/rRNA methyltransferase [*Klebsiella pneumoniae* subsp. *pneumoniae*]	OCN64920.1	123.18	30	6	0	24	0	ND	1.40E+03	9	1	0	8	39594
**Oxidative stress**														
heat shock protein 90 [*Klebsiella pneumoniae* subsp. *pneumoniae*]	AIW69779.1	217.79	54	8	5	50	9.46E+02	4.02E+02	7.72E+04	34	6	3	55	71093
catalase HPII [*Klebsiella pneumoniae* subsp. *pneumoniae*]	OCN92624.1	191.86	42	10	6	32	5.97E+03	3.86E+03	6.13E+04	33	7	7	44	83471
periplasmic chaperone [*Klebsiella pneumoniae* subsp. *pneumoniae*]	AIK80971.1	157.98	84	20	24	58	5.36E+02	4.05E+02	1.49E+05	19	4	4	27	16492
heat-shock protein Hsp20 [*Klebsiella pneumoniae* subsp. *pneumoniae*]	OCO77248.1	147.8	53	11	4	44	3.62E+03	2.11E+03	4.53E+04	11	6	1	15	21506
oxidative damage protection protein [*Klebsiella pneumoniae* subsp. *pneumoniae*]	OCO07259.1	126.51	63	20	20	53	3.22E+03	2.69E+02	1.07E+04	7	5	2	8	10919
Alkyl hydroperoxide reductase subunit F [*Klebsiella pneumoniae* subsp. *pneumoniae*]	KHF67361.1	122.86	16	0	0	16	ND	ND	6.09E+03	7	0	0	10	56019
OsmC family peroxiredoxin [*Klebsiella pneumoniae* subsp. *pneumoniae*]	OCO20669.1	119.42	30	9	0	21	4.87E+02	ND	1.11E+04	5	3	0	7	15121
cold-shock protein [*Klebsiella pneumoniae* subsp. *pneumoniae*]	AIX79865.1	111.77	67	35	19	42	8.90E+02	5.88E+02	2.65E+04	6	2	1	7	7402
superoxide dismutase [*Klebsiella pneumoniae* subsp. *pneumoniae*]	KPV67827.1	101.06	25	0	0	25	ND	ND	1.80E+04	5	0	0	12	22985
peptide-methionine (S)-S-oxide reductase [*Klebsiella pneumoniae* subsp. *pneumoniae*]	OCN33825.1	85.77	19	0	0	19	ND	ND	2.96E+03	3	0	0	8	23238
cold-shock protein [*Klebsiella pneumoniae* subsp. *pneumoniae*]	AIW70077.1	75.61	42	0	0	32	ND	ND	7.75E+04	4	0	0	12	7449
GTPase CgtA [*Klebsiella pneumoniae* subsp. *pneumoniae*]	AIW73264.1	71.87	6	0	0	4	ND	ND	2.56E+03	2	0	0	1	43577
universal stress global response regulator UspA [*Klebsiella pneumoniae* subsp. *pneumoniae*]	OCO25019.1	52.12	8	0	0	8	ND	ND	1.10E+03	2	0	0	2	16181
**Transcription and replication**														
elongation factor Tu [*Klebsiella pneumoniae* subsp. *pneumoniae*]	AIX82299.1	284.47	77	55	39	61	8.97E+04	8.44E+04	4.32E+05	87	73	63	158	43246
translation elongation factor G [*Klebsiella pneumoniae* subsp. *pneumoniae*]	OCN25432.1	250.81	56	6	4	50	1.24E+03	2.75E+02	1.87E+05	38	4	3	79	77543
30S ribosomal protein S2 [*Klebsiella pneumoniae* subsp. *pneumoniae*]	AIX67908.1	246.71	85	62	41	56	4.17E+04	1.03E+04	1.17E+05	54	51	19	53	26700
30S ribosomal protein S1 [*Klebsiella pneumoniae* subsp. *pneumoniae*]	OCN79247.1	219.27	48	6	4	41	3.58E+03	4.39E+03	1.45E+05	36	6	4	69	61177
30S ribosomal protein S6 [*Klebsiella pneumoniae* subsp. *pneumoniae*]	OCO17499.1	217.67	74	37	32	61	9.42E+03	1.65E+04	1.57E+05	16	9	11	29	15117
30S ribosomal protein S7 [*Klebsiella pneumoniae* subsp. *pneumoniae*]	AMA32523.1	203.78	77	70	51	58	4.48E+04	1.81E+04	1.48E+05	41	40	26	45	17618
DNA-directed RNA polymerase subunit beta' [*Klebsiella pneumoniae* subsp. *pneumoniae*]	AIX67235.1	199.4	28	1	1	23	9.39E+03	4.79E+03	6.82E+04	40	6	3	46	155262

DNA starvation/stationary phase protection protein Dps [*Klebsiella pneumoniae* subsp. *pneumoniae*]	OAK82820.1	196.08	66	21	10	58	1.38E+03	9.96E+02	1.00E+05	15	4	2	49	18708
ribosomal protein L1 [*Klebsiella pneumoniae* subsp. *pneumoniae*]	AIK81694.1	188.09	54	36	26	46	1.06E+04	3.05E+03	2.04E+05	25	26	8	40	24745
co-chaperone GroES [*Klebsiella pneumoniae* subsp. *pneumoniae*]	OCO29764.1	179.17	100	61	60	63	3.18E+04	3.08E+04	2.27E+05	20	18	28	30	10374
energy-dependent translational throttle protein EttA [*Klebsiella pneumoniae* subsp. *pneumoniae*]	OCN14146.1	177.25	34	0	0	34	ND	ND	4.78E+04	20	0	0	35	62237
protein disaggregation chaperone [*Klebsiella pneumoniae* subsp. *pneumoniae*]	AIX80448.1	176.88	36	1	0	30	3.23E+02	ND	1.84E+04	32	1	0	37	95406
trifunctional transcriptional regulator/proline dehydrogenase/L-glutamate gamma-semialdehyde dehydrogenase [*Klebsiella pneumoniae* subsp. *pneumoniae*]	OCN91910.1	174.71	22	3	2	15	1.12E+03	1.21E+03	3.08E+04	25	3	2	28	144589
DNA-directed RNA polymerase subunit alpha [*Klebsiella pneumoniae* subsp. *pneumoniae*]	OCN83525.1	171.85	45	12	18	38	8.90E+03	1.05E+04	1.14E+05	20	8	12	44	36466
transcription termination/antitermination protein NusA [*Klebsiella pneumoniae* subsp. *pneumoniae*]	OCN95974.1	164.68	28	0	4	26		1.73E+03	3.55E+04	18	0	3	27	54921
30S ribosomal protein S4 [*Klebsiella pneumoniae* subsp. *pneumoniae*]	OCN81596.1	164.53	53	15	16	42	1.45E+04	7.31E+03	1.28E+05	22	10	12	37	23502
DNA gyrase subunit B [*Klebsiella pneumoniae* subsp. *pneumoniae*]	AMA17435.1	145.76	20	0	2	16		2.44E+02	1.42E+04	18	0	2	19	90087
transcription termination factor Rho [*Klebsiella pneumoniae* subsp. *pneumoniae*]	OCO24746.1	143.89	44	8	9	33	3.67E+02	1.41E+03	1.04E+04	18	4	3	17	47022
transcription elongation factor GreA [*Klebsiella pneumoniae* subsp. *pneumoniae*]	OCN33342.1	135.72	68	0	0	66		ND	2.89E+04	11	0	0	16	17734
ribosome recycling factor [*Klebsiella pneumoniae* subsp. *pneumoniae*]	AIX67911.1	134.13	54	5	0	49	0	ND	3.34E+04	11	1	0	16	20631
RNA polymerase subunit sigma [*Klebsiella pneumoniae* subsp. *pneumoniae*]	KPV70863.1	126.5	16	2	2	16	5.79E+02	4.04E+02	1.07E+04	11	1	1	14	70153
DNA gyrase subunit A [*Klebsiella pneumoniae* subsp. *pneumoniae*]	AIW72207.1	124.78	12	0	0	9	ND	ND	5.50E+03	11	0	0	10	96982
RNA polymerase subunit sigma [*Klebsiella pneumoniae* subsp. *pneumoniae*]	AMV55823.1	33.18	6	0	0	6	ND	ND	7.06E+02	1	0	0	1	21720
RNA-binding transcriptional accessory protein [*Klebsiella pneumoniae*]	WP_060579283.1	111.69	14	0	0	13	ND	ND	2.22E+03	9	0	0	10	85158
DNA topoisomerase I [*Klebsiella pneumoniae* subsp. *pneumoniae*]	AIX69063.1	110.89	14	2	2	11	4.42E+02	6.11E+02	5.47E+03	11	1	2	8	97297
50S ribosomal protein L19 [*Klebsiella pneumoniae* subsp. *pneumoniae*]	KHF64514.1	107.91	57	0	0	57	ND	ND	2.99E+04	6	0	0	14	13133
DNA topoisomerase IV subunit A [*Klebsiella pneumoniae* subsp. *pneumoniae*]	OCN95834.1	105.91	10	0	0	8	ND	ND	2.77E+03	7	0	0	6	83620
arcA transcriptional dual regulator [*Klebsiella pneumoniae* subsp. *pneumoniae*]	AIK81172.1	101.48	24	0	0	20	ND	ND	1.84E+04	8	0	0	13	27272
RNA polymerase-binding protein DksA [*Klebsiella pneumoniae* subsp. *pneumoniae*]	OCO23566.1	100.58	26	8	8	18	2.07E+03	7.60E+02	2.65E+04	4	3	2	12	17486
transcriptional regulator Crp [*Klebsiella pneumoniae* subsp. *pneumoniae*]	OCO29161.1	99.69	27	0	0	27	ND	ND	9.04E+03	5	0	0	10	23656
transcription termination/antitermination protein NusG [*Klebsiella pneumoniae* subsp. *pneumoniae*]	OCO10424.1	97.11	19	0	0	19	ND	ND	6.51E+03	5	0	0	9	20546
transcriptional repressor protein MetJ [*Klebsiella pneumoniae* subsp. *pneumoniae*]	AMA22926.1	96.35	40	0	0	25	ND	ND	2.63E+03	5	0	0	5	12109

DNA polymerase I [*Klebsiella pneumoniae* subsp. *pneumoniae*]	OCN65064.1	93.69	6	0	0	6	ND	ND	2.14E+03	4	0	0	6	103211
MULTISPECIES: single-stranded DNA-binding protein [Gammaproteobacteria]	WP_032442031.1	92.29	28	0	0	24	ND	ND	8.58E+03	5	0	0	6	16701
DNA helicase UvrD [*Klebsiella pneumoniae* subsp. *pneumoniae*]	OYE95568.1	86.42	8	0	0	6	ND	ND	2.61E+02	4	0	0	4	84777
DNA-binding protein [*Klebsiella pneumoniae* subsp. *pneumoniae*]	AMA17066.1	83.44	20	0	0	15	ND	ND	2.11E+03	5	0	0	5	34428
DNA-binding response regulator [*Klebsiella pneumoniae* subsp. *pneumoniae*]	OCN87496.1	83.26	15	0	0	15	ND	ND	4.98E+03	4	0	0	8	26272
transcriptional regulator [*Klebsiella pneumoniae* subsp. *pneumoniae*]	OCN82748.1	82.22	26	0	0	16	ND	ND	2.00E+03	4	0	0	3	12429
ribonucleoside-diphosphate reductase subunit alpha [*Klebsiella pneumoniae* subsp. *pneumoniae*]	OCO47316.1	81.95	5	0	0	4	ND	ND	5.91E+02	5	0	0	6	85635
Transcriptional regulator SlyA [*Klebsiella pneumoniae* subsp. *pneumoniae*]	KHF52155.1	81.69	41	0	0	38	ND	ND	4.28E+03	6	0	0	6	16532
chromosomal replication initiator protein DnaA [*Klebsiella pneumoniae* subsp. *pneumoniae*]	AIK81916.1	74.06	9	0	0	7	ND	ND	9.26E+02	3	0	0	2	49718
DNA mismatch repair protein MutS [*Klebsiella pneumoniae* subsp. *pneumoniae*]	KHF64699.1	69.06	4	0	0	2	ND	ND	6.23E+02	3	0	0	1	95082
DNA-binding transcriptional regulator [*Klebsiella pneumoniae* subsp. *pneumoniae*]	OQZ17544.1	69.4	10	0	0	10	ND	ND	6.15E+02	2	0	0	3	34414
translation initiation factor IF-1 [*Klebsiella pneumoniae* subsp. *pneumoniae*]	KLA40800.1	69.3	39	0	0	39	ND	ND	1.04E+04	2	0	0	8	8250
transcriptional regulator [*Klebsiella pneumoniae* subsp. *pneumoniae*]	AMA29419.1	68.09	8	0	0	4	ND	ND	4.76E+02	2	0	0	1	44351
Holliday junction DNA helicase RuvA [*Klebsiella pneumoniae* subsp. *pneumoniae*]	OCN90319.1	49.73	6	0	0	6	ND	ND	1.07E+03	1	0	0	1	22129
ribosome-binding factor A [*Klebsiella pneumoniae* subsp. *pneumoniae*]	OCO20463.1	49.08	15	0	0	15	ND	ND	1.67E+03	2	0	0	2	15096
DNA topoisomerase IV [*Klebsiella pneumoniae* subsp. *pneumoniae*]	AMV50098.1	48.34	3	0	0	2	ND	ND	5.20E+02	2	0	0	1	70076
DEAD/DEAH family ATP-dependent RNA helicase [*Klebsiella pneumoniae* subsp. *pneumoniae*]	OCO07422.1	48.01	2	0	0	2	ND	ND	2.63E+02	1	0	0	1	72205
ribosomal RNA small subunit methyltransferase H [*Klebsiella pneumoniae* subsp. *pneumoniae*]	AMA28788.1	46.04	4	0	0	4	ND	ND	1.37E+03	1	0	0	1	34840
**Metabolism**														
phosphopyruvate hydratase [*Klebsiella pneumoniae* subsp. *pneumoniae*]	OCN79836.1	267.89	76	48	43	38	7.47E+04	6.03E+04	3.73E+05	69	76	58	77	45550
formate acetyltransferase [*Klebsiella pneumoniae* subsp. *pneumoniae*]	OCO37009.1	250.69	37	8	5	32	1.10E+04	4.79E+03	2.94E+05	45	17	8	83	85146
ATP synthase F0F1 subunit beta [*Klebsiella pneumoniae* subsp. *pneumoniae*]	AMV59494.1	215.38	60	20	32	47	6.86E+03	5.68E+03	1.63E+05	39	13	15	61	50210
dihydrolipoamide succinyltransferase [*Klebsiella pneumoniae* subsp. *pneumoniae*]	AIX78191.1	164.45	27	4	4	21	1.51E+03	2.70E+02	1.17E+05	15	2	2	35	44215
NAD(P)H:quinone oxidoreductase [*Klebsiella pneumoniae* subsp. *pneumoniae*]	AIW70461.1	151.48	33	14	14	25	1.16E+03	1.80E+02	7.84E+04	9	4	4	21	20910
bifunctional acetaldehyde-CoA/alcohol dehydrogenase [*Klebsiella pneumoniae* subsp. *pneumoniae*]	OCO06438.1	233.47	43	15	13	34	7.17E+03	4.25E+03	9.85E+04	46	20	15	57	95894

phosphoglycerate kinase [*Klebsiella pneumoniae* subsp. *pneumoniae*]	OCO12346.1	177.3	45	13	13	34	2.50E+03	5.69E+02	1.39E+05	26	8	5	40	41133
pyruvate dehydrogenase (acetyl-transferring) homodimeric type [*Klebsiella pneumoniae* subsp. *pneumoniae*]	OCN38258.1	190.51	32	0	0	28	ND	ND	8.48E+04	35	0	0	51	99434
F0F1 ATP synthase subunit alpha [*Klebsiella pneumoniae* subsp. *pneumoniae*]	OCN87778.1	148.56	30	5	0	19	1.57E+03	ND	9.05E+04	16	5	0	23	55169
ATP synthase F0 B subunit [*Klebsiella pneumoniae* subsp. *pneumoniae*]	AIK81889.1	181.96	66	29	18	66	4.99E+02	6.24E+02	9.53E+04	21	4	2	43	17130
Succinyl-CoA ligase [ADP-forming] subunit beta [*Klebsiella pneumoniae* subsp. *pneumoniae*]	KHF67283.1	192.4	44	15	7	38	4.07E+03	2.44E+03	8.73E+04	20	9	5	32	41503
transketolase [*Klebsiella pneumoniae* subsp. *pneumoniae*]	OCO19653.1	190.99	28	3	5	27	1.04E+03	1.29E+03	7.43E+04	24	2	3	49	71819
succinate--CoA ligase subunit alpha [*Klebsiella pneumoniae* subsp. *pneumoniae*]	OYF22683.1	194.11	51	25	19	43	8.16E+03	3.17E+03	7.78E+04	27	15	7	26	29867
phosphate acetyltransferase [*Klebsiella pneumoniae* subsp. *pneumoniae*]	AIK83354.1	185.53	28	2	0	27	3.03E+02	ND	6.93E+04	19	1	0	32	76637
succinate dehydrogenase flavoprotein subunit [*Klebsiella pneumoniae* subsp. *pneumoniae*]	AIK80411.1	192	36	0	0	33	ND	ND	4.27E+04	20	0	0	21	64478
bifunctional glucose-1-phosphatase/inositol phosphatase [*Klebsiella pneumoniae* subsp. *pneumoniae*]	OAK87121.1	153.7	33	0	0	25	ND	ND	3.38E+04	11	0	0	17	45518
2-oxoglutarate dehydrogenase E1 component [*Klebsiella pneumoniae* subsp. *pneumoniae*]	OCN40228.1	188.21	25	3	4	21	2.43E+03	1.18E+03	4.46E+04	24	5	5	34	105171
phosphoenolpyruvate carboxykinase [*Klebsiella pneumoniae* subsp. *pneumoniae*]	AMV54958.1	167.04	26	2	2	24	4.33E+02	0	4.12E+04	12	2	1	31	59577
fructose-bisphosphate aldolase [*Klebsiella pneumoniae* subsp. *pneumoniae*]	AIW72960.1	152.06	24	0	0	24	ND	ND	4.21E+04	9	0	0	18	39158
nitrate reductase [*Klebsiella pneumoniae* subsp. *pneumoniae*]	AIX84959.1	224	32	0	0	29	ND	ND	3.86E+04	37	0	0	57	140496
pyruvate dehydrogenase [*Klebsiella pneumoniae* subsp. *pneumoniae*]	KLA41713.1	171.08	31	2	0	30	2.25E+02	ND	4.06E+04	21	1	0	34	65916
trehalase family protein [*Klebsiella pneumoniae* subsp. *pneumoniae*]	AIK78777.1	189.73	28	5	5	26	1.33E+03	1.34E+03	4.11E+04	13	1	1	20	63155
pyruvate kinase [*Klebsiella pneumoniae* subsp. *pneumoniae*]	AMA30823.1	161.17	30	0	0	26	ND	ND	4.25E+04	16	0	0	26	50688
acetyl-coenzyme A synthetase [*Klebsiella pneumoniae* subsp. *pneumoniae*]	OYG08606.1	176.45	36	2	2	35	6.26E+02	6.41E+02	3.58E+04	22	1	1	31	71939
glucose-6-phosphate isomerase [*Klebsiella pneumoniae* subsp. *pneumoniae*]	OCO39405.1	198.24	42	0	0	35	ND	ND	2.29E+04	20	0	0	33	61328
aconitate hydratase [*Klebsiella pneumoniae* subsp. *pneumoniae*]	KLA40442.1	174.54	26	0	0	25	ND	ND	2.99E+04	20	0	0	31	97551
aconitate hydratase 2 [*Klebsiella pneumoniae* subsp. *pneumoniae*]	AIK81042.1	174.6	30	2	0	27	0	ND	2.40E+04	20	1	0	29	90665
phosphoenolpyruvate--protein phosphotransferase [*Klebsiella pneumoniae* subsp. *pneumoniae*]	OCO02796.1	160.3	34	3	3	26	0	0	2.33E+04	20	1	1	21	63268
catalase/peroxidase HPI [*Klebsiella pneumoniae* subsp. *pneumoniae*]	OCN24951.1	170.55	29	0	0	25	ND	ND	1.92E+04	21	0	0	23	78951
ATP-dependent protease ATP-binding subunit HslU [*Klebsiella pneumoniae* subsp. *pneumoniae*]	AIX67113.1	157.89	34	3	3	27	3.97E+02	5.91E+02	2.22E+04	15	1	1	12	49703
succinate dehydrogenase iron-sulfur subunit [*Klebsiella pneumoniae* subsp. *pneumoniae*]	AMA24241.1	126.41	42	0	0	29	ND	ND	1.52E+04	7	0	0	10	26724

acid phosphatase [*Klebsiella pneumoniae* subsp. *pneumoniae*]	AIW76180.1	184.31	51	0	0	39	ND	ND	2.26E+04	17	0	0	16	27006
acetyl-CoA carboxylase biotin carboxylase subunit [*Klebsiella pneumoniae* subsp. *pneumoniae]*	OCN96049.1	169.98	33	0	0	33	ND	ND	2.21E+04	14	0	0	22	49271
&beta-D-glucoside glucohydrolase periplasmic [*Klebsiella pneumoniae* subsp. *pneumoniae*]	AIK83480.1	152.65	22	0	0	17	ND	ND	1.41E+04	17	0	0	16	82341
nucleoside diphosphate kinase [*Klebsiella pneumoniae* subsp. *pneumoniae*]	AIW77965.1	126.4	38	0	0	38	ND	ND	2.09E+04	4	0	0	7	15526
phosphogluconate dehydrogenase (NADP(+)-dependent decarboxylating) [*Klebsiella pneumoniae* subsp. *pneumoniae*]	OAK88314.1	146.54	30	0	0	30	ND	ND	1.73E+04	15	0	0	24	51402
pyruvate kinase [*Klebsiella pneumoniae* subsp. *pneumoniae*]	AMV51230.1	159.47	36	0	0	36	ND	ND	1.98E+04	15	0	0	29	51422
fabB [*Klebsiella pneumoniae* subsp. *pneumoniae*]	AIK83327.1	107.95	18	0	0	16	ND	ND	2.00E+04	7	0	0	8	41598
glucan biosynthesis protein G [*Klebsiella pneumoniae* subsp. *pneumoniae*]	OCN42171.1	156.96	31	2	2	26	2.68E+02	3.78E+02	1.91E+04	16	2	1	22	58570
inorganic pyrophosphatase [*Klebsiella pneumoniae* subsp. *pneumoniae*]	OCO17533.1	97.9	27	0	0	22	ND	ND	1.04E+04	5	0	0	6	19721
enoyl-[acyl-carrier-protein] reductase [*Klebsiella pneumoniae* subsp. *pneumoniae*]	OCN93341.1	117.02	23	0	0	23	ND	ND	1.02E+04	7	0	0	10	27911
mannose-6-phosphate isomerase [*Klebsiella pneumoniae* subsp. *pneumoniae*]	AMV57235.1	109.42	20	0	0	20	ND	ND	1.27E+04	6	0	0	9	42395
phosphoenolpyruvate synthase [*Klebsiella pneumoniae* subsp. *pneumoniae*]	AIW71634.1	128.05	16	0	0	11	ND	ND	7.20E+03	12	0	0	12	87061
Dimethyl sulfoxide reductase DmsA precursor [*Klebsiella pneumoniae* subsp. *pneumoniae*]	AKE76777.1	152.83	25	2	2	17	7.82E+01	0	9.70E+03	18	1	1	14	89985
NADH dehydrogenase [*Klebsiella pneumoniae* subsp. *pneumoniae*]	KPV70450.1	106.76	28	0	0	28	ND	ND	9.29E+03	4	0	0	8	18561
gamma-glutamyltranspeptidase [*Klebsiella pneumoniae* subsp. *pneumoniae*]	AMA32604.1	153.48	30	0	0	20	ND	ND	9.76E+03	15	0	0	14	62009
acyl-CoA thioesterase [*Klebsiella pneumoniae* subsp. *pneumoniae*]	OCO04334.1	126.49	21	0	0	21	ND	ND	7.57E+03	8	0	0	9	45543
leucyl aminopeptidase [*Klebsiella pneumoniae* subsp. *pneumoniae*]	OCN23711.1	96.56	12	0	0	10	ND	ND	5.78E+03	7	0	0	8	54848
Phosphoglucosamine mutase [*Klebsiella pneumoniae* subsp. *pneumoniae*]	KHF65227.1	109.83	14	0	0	12	ND	ND	4.60E+03	6	0	0	10	47905
PTS system trehalose(maltose)-specific transporter subunits IIBC [*Klebsiella pneumoniae* subsp. *pneumoniae*]	KLA42042.1	95.6	11	0	0	9	ND	ND	5.50E+03	5	0	0	6	50895
quinone oxidoreductase [*Klebsiella pneumoniae* subsp. *pneumoniae*]	OCN82190.1	105.2	22	0	0	11	ND	ND	3.36E+03	6	0	0	3	34509
glucosamine--fructose-6-phosphate aminotransferase [*Klebsiella pneumoniae* subsp. *pneumoniae]*	AIX82914.1	101.29	15	0	0	12	ND	ND	4.73E+03	5	0	0	4	38478
3-hydroxydecanoyl-ACP dehydratase [*Klebsiella pneumoniae* subsp. *pneumoniae*]	AIX83780.1	70.78	21	0	0	21	ND	ND	7.20E+03	3	0	0	6	19084
1 4-dihydroxy-2-naphthoyl-CoA synthase [*Klebsiella pneumoniae* subsp. *pneumoniae*]	OCN66319.1	68.66	19	0	0	16	ND	ND	2.48E+03	5	0	0	4	31625
Fe-S cluster assembly scaffold IscU [*Klebsiella pneumoniae* subsp. *pneumoniae*]	OCO37561.1	98.5	47	0	0	36	ND	ND	5.07E+03	6	0	0	8	13807
maltose phosphorylase [*Klebsiella pneumoniae* subsp. *pneumoniae*]	AMV54944.1	91.58	8	0	0	6	ND	ND	1.88E+02	7	0	0	5	90059

PTS system N-acetylglucosamine-specific IIBC component [*Klebsiella pneumoniae* subsp. *pneumoniae*]	AIK80444.1	107.22	11	0	0	11	ND	ND	1.22E+03	4	0	0	9	66031
gpt [*Klebsiella pneumoniae* subsp. *pneumoniae*]	AIK80920.1	100.58	24	0	0	16	ND	ND	2.34E+03	3	0	0	2	16935
glucose-6-phosphate dehydrogenase [*Klebsiella pneumoniae* subsp. *pneumoniae*]	OCN83458.1	91.38	13	0	0	12	ND	ND	3.19E+03	7	0	0	6	55872
keto-deoxy-phosphogluconate aldolase [*Klebsiella pneumoniae* subsp. *pneumoniae]*	OCN99860.1	88.76	26	0	0	26	ND	ND	2.35E+03	4	0	0	4	22229
glutathione reductase [*Klebsiella pneumoniae* subsp. *pneumoniae*]	AIX81431.1	64.07	6	0	0	3	ND	ND	1.22E+03	2	0	0	1	48662
glucosamine--fructose-6-phosphate aminotransferase [*Klebsiella pneumoniae* subsp. *pneumoniae*]	AMV58850.1	84.85	15	0	0	15	ND	ND	2.07E+03	5	0	0	5	39163
type I methionyl aminopeptidase [*Klebsiella pneumoniae* subsp. *pneumoniae*]	OCN59504.1	56.4	9	0	0	9	ND	ND	1.68E+03	2	0	0	2	29179
Bifunctional phosphopantothenoylcysteine decarboxylase/phosphopantothenate synthase [*Klebsiella pneumoniae* subsp. *pneumoniae*]	OCN80852.1	67.49	6	0	0	6	ND	ND	1.68E+03	2	0	0	3	43334
acyl-CoA dehydrogenase [*Klebsiella pneumoniae* subsp. *pneumoniae*]	OCO31480.1	52.48	2	0	0	1	ND	ND	3.81E+02	2	0	0	1	89218
glucokinase [*Klebsiella pneumoniae* subsp. *pneumoniae*]	OCO26273.1	95.52	19	0	0	19	ND	ND	4.51E+03	6	0	0	10	34422
glutamate synthase [*Klebsiella pneumoniae* subsp. *pneumoniae*]	OCN46160.1	53.83	4	0	0	4	ND	ND	9.53E+02	1	0	0	1	51910
cobalamin biosynthesis protein CobQ [*Klebsiella pneumoniae* subsp. *pneumoniae*]	AIX80676.1	75.6	6	0	0	6	ND	ND	3.98E+02	2	0	0	4	55028
mannonate dehydratase [*Klebsiella pneumoniae* subsp. *pneumoniae*]	OCO38164.1	62.64	8	0	0	7	ND	ND	9.39E+02	3	0	0	2	44770
peptide deformylase [*Klebsiella pneumoniae* subsp. *pneumoniae*]	OCO11101.1	61.36	8	8	0	7	3.25E+02	ND	1.74E+03	2	1	0	2	19355
precorrin-8X methylmutase [*Klebsiella pneumoniae* subsp. *pneumoniae*]	OCN68790.1	51.42	6	0	0	6	ND	ND	4.30E+02	1	0	0	1	22846
3-oxoacyl-ACP synthase [*Klebsiella pneumoniae* subsp. *pneumoniae*]	AIW70520.1	69.88	8	0	0	8	ND	ND	2.84E+03	2	0	0	2	33422
methenyltetrahydrofolate cyclohydrolase [*Klebsiella pneumoniae* subsp. *pneumoniae*]	AIW75356.1	70.98	10	0	0	10	ND	ND	4.12E+03	2	0	0	3	30974
glucose dehydrogenase [Klebsiella pneumoniae subsp. pneumoniae]	OAK88172.1	179.39	27	0	0	27	ND	ND	1.84E+04	19	0	0	31	86422
diaminobutyrate--2-oxoglutarate transaminase [*Klebsiella pneumoniae* subsp. *pneumoniae*]	OCO41693.1	107.45	21	0	0	18	ND	ND	5.54E+03	8	0	0	8	49792
**Transport**														
ABC transporter substrate-binding protein [*Klebsiella pneumoniae* subsp. *pneumoniae*]	ROE44419.1	206.34	56	3	3	52	1.11E+03	9.22E+02	9.56E+04	22	2	2	48	39565
glutamate/aspartate ABC transporter substrate-binding protein [*Klebsiella pneumoniae* subsp. *pneumoniae*]	OCN87377.1	212.34	71	16	10	61	4.36E+03	4.83E+03	8.05E+04	30	5	4	49	33472
putative sugar ABC transporter [*Klebsiella pneumoniae* subsp. *pneumoniae*]	AIK82512.1	146.34	36	0	4	30	ND	0	6.83E+04	11	0	1	22	35219
methyl-galactoside ABC transporter substrate-binding protein [*Klebsiella pneumoniae* subsp. *pneumoniae*]	OCO36175.1	194.99	65	0	0	59	ND	ND	3.27E+04	21	0	0	36	35831
ATPase (plasmid) [Klebsiella pneumoniae subsp. pneumoniae]	OQZ55785.1	206.72	42	7	7	37	6.36E+03	4.71E+03	4.80E+04	44	6	11	56	102413

sugar ABC transporter substrate-binding protein [*Klebsiella pneumoniae* subsp. *pneumoniae*]	AIW74420.1	173.03	46	0	0	46	ND	ND	3.04E+04	19	0	0	34	43257
dppA [*Klebsiella pneumoniae* subsp. *pneumoniae*]	AIK82123.1	150.16	29	0	0	24	ND	ND	3.25E+04	16	0	0	19	59212
translocation protein TolB [*Klebsiella pneumoniae* subsp. *pneumoniae*]	KLA41000.1	162.54	32	0	8	25	ND	0	2.14E+04	10	0	1	14	45912
glutamine ABC transporter substrate-bindnig protein [*Klebsiella pneumoniae* subsp. *pneumoniae*]	AIX68599.1	157.34	30	0	0	30	ND	ND	2.36E+04	10	0	0	22	27152
spermidine/putrescine ABC transporter substrate-binding protein [*Klebsiella pneumoniae* subsp. *pneumoniae*]	AMV57605.1	132.97	32	0	0	32	ND	ND	2.48E+04	11	0	0	22	38873
ferrous iron transporter [*Klebsiella pneumoniae* subsp. *pneumoniae*]	KPV68208.1	163.12	41	0	0	38	ND	ND	1.89E+04	13	0	0	16	41012
preprotein translocase SecG subunit [*Klebsiella pneumoniae* subsp. *pneumoniae*]	AIK82442.1	81.91	33	0	0	33	ND	ND	1.22E+04	2	0	0	7	9091
DL-methionine transporter substrate-binding subunit [*Klebsiella pneumoniae* subsp. *pneumoniae*]	OCN79173.1	150.18	42	0	0	42	ND	ND	1.37E+04	8	0	0	14	29384
glutathione ABC transporter substrate-binding protein [*Klebsiella pneumoniae* subsp. *pneumoniae*]	AIX83668.1	132.25	22	0	0	22	ND	ND	1.29E+04	10	0	0	17	56446
spermidine/putrescine ABC transporter substrate-binding protein [*Klebsiella pneumoniae* subsp. *pneumoniae*]	KLA39781.1	123.1	38	0	0	35	ND	ND	9.83E+03	12	0	0	13	42220
nucleoside-specific channel-forming protein Tsx [*Klebsiella pneumoniae* subsp. *pneumoniae*]	OCO21523.1	97.08	16	0	0	8	ND	ND	9.98E+03	4	0	0	6	33507
phosphate ABC transporter phosphate-binding protein PstS [*Klebsiella pneumoniae* subsp. *pneumoniae*]	AIK81899.1	137.21	44	5	5	44	2.31E+02	0	9.18E+03	11	2	1	14	34983
amino acid ABC transporter substrate-binding protein [*Klebsiella pneumoniae* subsp. *pneumoniae*]	OYE09813.1	147.18	48	0	0	48	ND	ND	9.69E+03	9	0	0	16	27566
spermidine/putrescine ABC transporter substrate-binding protein [*Klebsiella pneumoniae* subsp. *pneumoniae*]	AIX78376.1	107.2	23	0	0	23	ND	ND	3.68E+03	7	0	0	9	40836
PTS glucose transporter subunit IIBC [*Klebsiella pneumoniae* subsp. *pneumoniae*]	OCO12056.1	96.08	11	0	0	11	ND	ND	5.77E+03	5	0	0	5	50655
histidine ABC transporter substrate-binding protein HisJ [*Klebsiella pneumoniae* subsp. *pneumoniae*]	OCO36067.1	109.8	27	0	0	27	ND	ND	6.36E+03	7	0	0	8	28547
PTS system mannose/fructose/sorbose IID component family protein [*Klebsiella pneumoniae* subsp. *pneumoniae*]	AIK81222.1	75.6	10	0	0	6	ND	ND	5.31E+03	2	0	0	2	31054
D-ribose ABC transporter substrate-binding protein RbsB [*Klebsiella pneumoniae* subsp. *pneumoniae*]	OAK84018.1	123.09	35	0	0	35	ND	ND	4.02E+03	8	0	0	9	31017
PTS mannitol transporter subunit IICBA [*Klebsiella pneumoniae* subsp. *pneumoniae*]	OCN90157.1	82.6	6	0	0	6	ND	ND	5.07E+03	3	0	0	5	67733
PTS mannose transporter subunit IID [*Klebsiella pneumoniae* subsp. *pneumoniae*]	AIX85064.1	70.25	15	4	4	12	3.25E+02	0	3.55E+03	4	2	2	5	30912
spermidine/putrescine ABC transporter [*Klebsiella pneumoniae* subsp. *pneumoniae*]	AMV51859.1	69.21	7	0	0	7	ND	ND	2.00E+03	2	0	0	5	40859
preprotein translocase YajC subunit [*Klebsiella pneumoniae* subsp. *pneumoniae*]	AIK80801.1	64.93	27	0	0	27	ND	ND	3.33E+03	3	0	0	4	11859
PTS fructose transporter subunit IIBC [*Klebsiella pneumoniae* subsp. *pneumoniae*]	OCN89527.1	106.23	12	0	0	12	ND	ND	2.98E+03	5	0	0	6	57549
secretion protein HlyD [*Klebsiella pneumoniae* subsp. *pneumoniae*]	OCO05503.1	92.81	16	0	0	12	ND	ND	2.03E+03	4	0	0	3	36014
molybdate transporter [*Klebsiella pneumoniae* subsp. *pneumoniae*]	AIW75713.1	103.19	25	0	0	20	ND	ND	2.07E+03	5	0	0	7	26949

ytfQ [*Klebsiella pneumoniae* subsp. *pneumoniae*]	AIK81432.1	109.04	28	0	6	20	ND	0	4.55E+03	8	0	1	6	32291
sulfate ABC transporter substrate-binding protein [*Klebsiella pneumoniae* subsp. *pneumoniae*]	AMV59446.1	86.34	29	0	0	29	ND	ND	5.48E+03	4	0	0	7	21340
HAAAP family serine/threonine permease partial [*Klebsiella pneumoniae*]	WP_064185871.1	62.88	4	0	0	4	ND	ND	3.62E+03	1	0	0	4	41208
DL-methionine transporter substrate-binding subunit [*Klebsiella pneumoniae* subsp. *pneumoniae*]	OCN23110.1	54.43	9	0	0	9	ND	ND	1.13E+03	2	0	0	2	29100
efflux transporter RND family MFP subunit [*Klebsiella pneumoniae* subsp. *pneumoniae*]	AIK80585.1	76.13	8	0	0	8	ND	ND	1.26E+03	2	0	0	2	38820
magnesium-translocating P-type ATPase [*Klebsiella pneumoniae* subsp. *pneumoniae*]	AMV59013.1	70.75	4	0	0	3	ND	ND	8.96E+02	3	0	0	3	99909
magnesium and cobalt transport protein CorA [*Klebsiella pneumoniae* subsp. *pneumoniae*]	OCN97568.1	40.54	3	0	0	3	ND	ND	4.27E+02	1	0	0	1	36668
peptide ABC transporter ATP-binding protein [*Klebsiella pneumoniae* subsp. *pneumoniae*]	AMA17637.1	59.15	7	0	0	7	ND	ND	9.18E+02	2	0	0	2	35764
ligand-gated channel protein [*Klebsiella pneumoniae* subsp. *pneumoniae*]	OCN28670.1	76.92	7	0	0	3	ND	ND	1.10E+02	3	0	0	1	80474
protein TolQ [*Klebsiella pneumoniae* subsp. *pneumoniae*]	OCO24408.1	64.16	13	0	0	13	ND	ND	1.53E+03	2	0	0	4	25269
PTS lactose transporter subunit IIA [*Klebsiella pneumoniae* subsp. *pneumoniae*]	OCN80386.1	54.39	24	0	0	24	ND	ND	7.74E+02	2	0	0	2	11538
glutamine ABC transporter ATP-binding protein [*Klebsiella pneumoniae* subsp. *pneumoniae*]	OCN81129.1	77.94	15	0	0	15	ND	ND	1.22E+03	3	0	0	4	26677
D-serine/D-alanine/glycine transporter [*Klebsiella pneumoniae* subsp. *pneumoniae*]	OCO34338.1	60	6	0	0	6	ND	ND	7.98E+02	2	0	0	2	51234
ABC transporter substrate-binding protein [*Klebsiella pneumoniae* subsp. *pneumoniae*]	AMA16224.1	70.13	13	0	0	10	ND	ND	4.82E+02	4	0	0	3	41338
sugar ABC transporter substrate-binding protein [*Klebsiella pneumoniae* subsp. *pneumoniae*]	RNX94183.1	57.93	31	0	0	16	ND	ND	7.93E+02	2	0	0	1	9855
ABC transporter substrate-binding protein [*Klebsiella pneumoniae* subsp. *pneumoniae*]	OQZ37576.1	74.4	13	0	0	8	ND	ND	3.76E+02	3	0	0	3	33182
ABC transporter substrate-binding protein [*Klebsiella pneumoniae* subsp. *pneumoniae*]	AIX78043.1	92.24	9	0	0	9	ND	ND	3.33E+02	3	0	0	5	56860
heme ABC exporter ATP-binding protein CcmA [*Klebsiella pneumoniae* subsp. *pneumoniae*]	AIK80296.1	49.92	4	0	0	2	ND	ND	3.80E+02	2	0	0	1	58852
**Nucleotide metabolism**														
2' 3'-cyclic-nucleotide 2'-phosphodiesterase [*Klebsiella pneumoniae* subsp. *pneumoniae*]	OYF13578.1	162.91	30	0	0	30	ND	ND	1.37E+04	16	0	0	27	70585
purine nucleoside phosphorylase [*Klebsiella pneumoniae* subsp. *pneumoniae*]	AIK81188.1	92.06	20	0	0	14	ND	ND	1.36E+04	4	0	0	4	24974
uracil phosphoribosyltransferase [*Klebsiella pneumoniae* subsp. *pneumoniae*]	OCO31827.1	107.9	34	0	0	30	ND	ND	9.33E+03	7	0	0	10	22565
nucleotide-binding protein [*Klebsiella pneumoniae* subsp. *pneumoniae*]	KPV68913.1	124.14	55	0	0	52	ND	ND	9.87E+03	10	0	0	9	18335
Uridine phosphorylase [*Klebsiella pneumoniae* subsp. *pneumoniae*]	KHF63849.1	114.75	21	0	0	21	ND	ND	1.18E+04	8	0	0	14	27053
bifunctional UDP-sugar hydrolase/5'-nucleotidase [*Klebsiella pneumoniae* subsp. *pneumoniae*]	OYG24635.1	114.92	17	0	0	17	ND	ND	1.07E+04	7	0	0	11	60522

Adenylosuccinate synthetase [*Klebsiella pneumoniae* subsp. *pneumoniae*]	AKE77991.1	113.74	26	0	0	20	ND	ND	6.63E+03	10	0	0	8	47166
exoribonuclease R [*Klebsiella pneumoniae* subsp. *pneumoniae*]	AIW74585.1	111.87	12	0	0	11	ND	ND	4.84E+03	10	0	0	10	91816
DUF1338 domain-containing protein [*Klebsiella pneumoniae* subsp. *pneumoniae*]	OCN88640.1	93.52	14	0	0	14	ND	ND	7.24E+03	5	0	0	12	50715
oxidoreductase [*Klebsiella pneumoniae* subsp. *pneumoniae*]	AMA29062.1	96.13	23	0	0	15	ND	ND	2.25E+03	6	0	0	7	36155
excinuclease ABC subunit A [*Klebsiella pneumoniae* subsp. *pneumoniae*]	AIW68898.1	108.87	12	0	0	8	ND	ND	2.01E+03	9	0	0	6	103974
putative oxidoreductase [*Klebsiella pneumoniae* subsp. *pneumoniae*]	AIK82919.1	88.63	20	0	0	16	ND	ND	3.28E+03	7	0	0	6	49977
ribonuclease E activity regulator RraA [*Klebsiella pneumoniae* subsp. *pneumoniae*]	AMA28070.1	71.19	13	0	0	13	ND	ND	3.01E+03	3	0	0	3	17385
nucleoside triphosphate hydrolase [*Klebsiella pneumoniae* subsp. *pneumoniae*]	OCN40187.1	84.46	12	0	0	8	ND	ND	1.39E+03	4	0	0	2	39205
exodeoxyribonuclease III [*Klebsiella pneumoniae* subsp. *pneumoniae*]	OCN92642.1	78.33	18	0	0	14	ND	ND	1.08E+03	4	0	0	3	30898
ribonuclease PH [*Klebsiella pneumoniae* subsp. *pneumoniae*]	OCO29412.1	62.35	16	0	0	16	ND	ND	1.29E+03	3	0	0	4	25270
**Protein and amino acid**														
peptidyl-prolyl cis-trans isomerase in protein folding [*Klebsiella pneumoniae* subsp. *pneumoniae*]	AIK82297.1	151.19	55	5	0	41	0	ND	6.34E+04	15	1	0	18	29434
histidine ammonia-lyase [*Klebsiella pneumoniae* subsp. *pneumoniae*]	AMA29505.1	168.76	32	0	0	27	ND	ND	1.80E+04	13	0	0	21	53904
PrkA family serine protein kinase [*Klebsiella pneumoniae* subsp. *pneumoniae*]	OCN92669.1	181.7	28	6	2	24	1.46E+03	0	2.77E+04	17	3	1	31	74390
alanine--tRNA ligase [*Klebsiella pneumoniae* subsp. *pneumoniae*]	OCO19336.1	199.02	30	0	1	28	ND	0	2.27E+04	23	0	1	33	95622
ATP-dependent metalloprotease [*Klebsiella pneumoniae* subsp. *pneumoniae*]	AIX71335.1	189.37	38	2	4	31	2.51E+02	6.92E+02	2.82E+04	24	2	3	31	70781
glycine--tRNA ligase subunit beta [*Klebsiella pneumoniae* subsp. *pneumoniae*]	AMA32722.1	167.82	35	0	0	30	ND	ND	2.29E+04	22	0	0	25	76331
serine--tRNA ligase [*Klebsiella pneumoniae* subsp. *pneumoniae*]	OCO08422.1	167.15	35	15	5	20	1.26E+03	5.15E+02	2.39E+04	20	8	4	23	48603
valine--tRNA ligase [*Klebsiella pneumoniae* subsp. *pneumoniae*]	OCO30437.1	162.02	18	0	0	18	ND	ND	2.00E+04	16	0	0	22	108294
lysine decarboxylase LdcC [*Klebsiella pneumoniae* subsp. *pneumoniae*]	AIX83319.1	165.79	21	3	0	17	8.08E+02	ND	1.04E+04	15	2	0	16	81386
Clp protease ClpX [*Klebsiella pneumoniae* subsp. *pneumoniae*]	AIW75269.1	146.82	41	3	3	37	7.39E+02	4.12E+02	1.45E+04	16	2	1	23	46294
HflK protein [*Klebsiella pneumoniae* subsp. *pneumoniae*]	OCO69007.1	141.33	36	0	0	30	ND	ND	1.94E+04	13	0	0	16	45582
serine-type D-Ala-D-Ala carboxypeptidase [*Klebsiella pneumoniae* subsp. *pneumoniae*]	OCO67202.1	141.64	44	3	3	32	2.88E+02	0	1.76E+04	14	1	1	18	43491
aromatic amino acid aminotransferase [*Klebsiella pneumoniae* subsp. *pneumoniae*]	OCO23088.1	140.29	34	0	0	32	ND	ND	1.55E+04	15	0	0	18	43539
leucine--tRNA ligase [*Klebsiella pneumoniae* subsp. *pneumoniae*]	OCN94580.1	137.37	17	0	0	13	ND	ND	1.22E+04	15	0	0	17	97026
proline--tRNA ligase [*Klebsiella pneumoniae* subsp. *pneumoniae*]	AMA23746.1	130.62	18	0	0	14	ND	ND	1.21E+04	9	0	0	10	63524

peptidylprolyl isomerase SurA [*Klebsiella pneumoniae* subsp. *pneumoniae*]	OCN80433.1	138.17	38	0	0	23	ND	ND	1.21E+04	13	0	0	13	47062
asparagine--tRNA ligase [*Klebsiella pneumoniae* subsp. *pneumoniae*]	OCN87665.1	138.29	27	3	3	23	0	3.25E+02	1.27E+04	14	1	1	17	52495
preprotein translocase subunit SecA [*Klebsiella pneumoniae* subsp. *pneumoniae*]	AMA23642.1	163.41	17	0	0	13	ND	ND	1.40E+04	17	0	0	21	101991
elongation factor P [*Klebsiella pneumoniae* subsp. *pneumoniae*]	OCO21153.1	97.73	26	0	12	19	ND	7.97E+01	7.82E+03	7	0	2	6	20603
tryptophanyl-tRNA synthetase [*Klebsiella pneumoniae* subsp. *pneumoniae*]	AMV54975.1	103	18	0	0	18	ND	ND	9.79E+03	8	0	0	13	37533
histidine--tRNA ligase [*Klebsiella pneumoniae* subsp. *pneumoniae*]	AIK83190.1	112.72	17	0	0	17	ND	ND	9.32E+03	6	0	0	9	45973
methionine--tRNA ligase [*Klebsiella pneumoniae* subsp. *pneumoniae*]	AMA19261.1	111.28	15	0	0	15	ND	ND	7.16E+03	8	0	0	11	76165
serine dehydratase [*Klebsiella pneumoniae* subsp. *pneumoniae*]	AIX80646.1	107.16	13	0	0	11	ND	ND	6.71E+03	6	0	0	8	48487
hflC protein [*Klebsiella pneumoniae* subsp. *pneumoniae*]	AIK81480.1	109.89	31	0	0	31	ND	ND	3.21E+03	8	0	0	14	37615
Tyrosine--tRNA ligase [*Klebsiella pneumoniae* subsp. *pneumoniae*]	AKE75735.1	127.59	23	0	0	21	ND	ND	6.18E+03	10	0	0	11	47250
aminopeptidase PepB [*Klebsiella pneumoniae* subsp. *pneumoniae*]	OCN89625.1	132.54	25	0	0	25	ND	ND	5.65E+03	10	0	0	13	46219
glutamate--tRNA ligase [*Klebsiella pneumoniae* subsp. *pneumoniae*]	OCN67251.1	109.58	16	0	3	15	ND	2.25E+02	5.67E+03	8	0	1	7	53572
aspartate--tRNA ligase [*Klebsiella pneumoniae* subsp. *pneumoniae*]	AIK78695.1	92.23	13	0	0	9	ND	ND	3.85E+03	6	0	0	6	64444
lysine--tRNA ligase [*Klebsiella pneumoniae* subsp. *pneumoniae*]	OCN88486.1	158.29	41	4	0	32	ND	ND	5.25E+03	20	1	0	16	57652
ATP-dependent Clp protease ATP-binding subunit ClpA [*Klebsiella pneumoniae* subsp. *pneumoniae*]	OCO04286.1	118.61	16	7	2	6	9.83E+02	5.20E+02	2.05E+03	11	5	1	5	84156
protein-export membrane protein SecF [*Klebsiella pneumoniae* subsp. *pneumoniae*]	AIK80799.1	105.21	19	0	0	19	ND	ND	2.53E+03	4	0	0	7	35489
Phenylalanine--tRNA ligase alpha subunit [*Klebsiella pneumoniae* subsp. *pneumoniae*]	AKE75531.1	109.18	28	0	0	19	ND	ND	3.47E+03	8	0	0	7	36799
peptidase T [*Klebsiella pneumoniae* subsp. *pneumoniae*]	OCN63458.1	90.13	12	0	0	12	ND	ND	4.59E+03	4	0	0	7	44917
cysteine--tRNA ligase [*Klebsiella pneumoniae* subsp. *pneumoniae*]	OCN98823.1	114.31	11	0	0	11	ND	ND	9.43E+03	5	0	0	7	52168
Protease 4 [*Klebsiella pneumoniae* subsp. *pneumoniae*]	AKE76501.1	85.4	9	0	0	2	ND	ND	5.30E+02	4	0	0	1	66807
arginine decarboxylase [*Klebsiella pneumoniae* subsp. *pneumoniae*]	OCO28778.1	84.51	10	0	0	3	ND	ND	3.14E+03	5	0	0	4	73798
ATP-dependent Clp endopeptidase proteolytic subunit ClpP [*Klebsiella pneumoniae* subsp. *pneumoniae*]	AIK80759.1	81.83	19	0	0	19	ND	ND	5.88E+03	3	0	0	6	21692
L-serine ammonia-lyase [*Klebsiella pneumoniae* subsp. *pneumoniae*]	OCN62452.1	102.62	11	0	0	11	ND	ND	2.57E+03	4	0	0	7	48996
5'-methylthioadenosine nucleosidase [*Klebsiella pneumoniae* subsp. *pneumoniae*]	AIX77566.1	48.16	8	0	0	5	ND	ND	3.27E+03	2	0	0	1	24504
glycine--tRNA ligase subunit alpha [*Klebsiella pneumoniae* subsp. *pneumoniae*]	OCN45044.1	68.86	9	0	0	9	ND	ND	1.88E+03	2	0	0	2	34732
Arginine--tRNA ligase [*Klebsiella pneumoniae* subsp. *pneumoniae*]	KHF66828.1	98.54	12	0	0	12	ND	ND	2.41E+03	5	0	0	9	64243

arginine ABC transporter substrate-binding protein [*Klebsiella pneumoniae* subsp. *pneumoniae*]	OCO04304.1	79.16	22	0	0	16	ND	ND	3.02E+03	4	0	0	3	26799
succinylarginine dihydrolase [*Klebsiella pneumoniae* subsp. *pneumoniae*]	OCO66424.1	94.98	22	9	4	11	2.38E+02	0	1.26E+03	6	2	1	4	49456
acetylornithine aminotransferase [*Klebsiella pneumoniae* subsp. *pneumoniae*]	OCO05138.1	113.79	14	0	0	14	ND	ND	1.88E+03	4	0	0	4	43507
molecular chaperone [*Klebsiella pneumoniae* subsp. *pneumoniae*]	OCN86134.1	36.7	7	0	0	7	ND	ND	7.86E+02	1	0	0	1	20596
Cytidylate kinase [*Klebsiella pneumoniae* subsp. *pneumoniae*]	KHF67696.1	70.19	22	0	0	18	ND	ND	1.17E+03	4	0	0	3	24874
disulfide bond formation protein DsbA [*Klebsiella pneumoniae* subsp. *pneumoniae*]	OCN12504.1	62.44	10	0	0	10	ND	ND	2.41E+03	2	0	0	5	27691
gamma-glutamyl kinase [*Klebsiella pneumoniae* subsp. *pneumoniae*]	AIX77649.1	61.19	9	0	0	9	ND	ND	5.67E+02	2	0	0	2	39117
branched-chain amino acid aminotransferase [*Klebsiella pneumoniae* subsp. *pneumoniae*]	OCN19633.1	59.51	9	0	0	9	ND	ND	1.17E+03	2	0	0	2	33912
methionine synthase [*Klebsiella pneumoniae* subsp. *pneumoniae*]	AMA23074.1	48.13	1	0	0	1	ND	ND	7.09E+02	1	0	0	1	135813
asparagine synthase B [*Klebsiella pneumoniae* subsp. *pneumoniae*]	AMA24203.1	40.92	2	0	0	2	ND	ND	4.26E+02	1	0	0	1	62458
histidine ABC transporter substrate-binding protein HisJ [*Klebsiella pneumoniae* subsp. *pneumoniae*]	AIX80244.1	55.61	12	0	0	5	ND	ND	6.78E+02	2	0	0	1	28124
phenylalanine--tRNA ligase subunit beta partial [*Klebsiella pneumoniae* subsp. *pneumoniae*]	KPV67968.1	68.66	12	0	0	6	ND	ND	2.02E+03	5	0	0	3	50513
protease [*Klebsiella pneumoniae* subsp. *pneumoniae*]	AMA26690.1	66.2	6	0	0	4	ND	ND	1.21E+03	5	0	0	3	107410
queuine tRNA-ribosyltransferase [*Klebsiella pneumoniae* subsp. *pneumoniae*]	AIW69680.1	59.36	12	0	0	7	ND	ND	5.15E+02	3	0	0	2	42568
**Cell cycle**														
cell division protein FtsZ [*Klebsiella pneumoniae* subsp. *pneumoniae*]	OCO07866.1	191.71	43	7	9	40	9.52E+02	0	2.44E+04	16	3	2	30	40342
septum site-determining protein MinD [*Klebsiella pneumoniae* subsp. *pneumoniae*]	OCN62462.1	172.28	46	25	20	42	4.00E+03	3.86E+03	2.22E+04	16	8	7	18	29659
cell division protein DamX [*Klebsiella pneumoniae* subsp. *pneumoniae*]	OYE84427.1	171.86	39	0	0	33	ND	ND	2.52E+04	15	0	0	22	45776
cell division protein ZipA [*Klebsiella pneumoniae* subsp. *pneumoniae*]	OCN92064.1	157.92	24	0	0	24	ND	ND	1.34E+04	6	0	0	9	39203
GTP-binding protein TypA [*Klebsiella pneumoniae* subsp. *pneumoniae*]	OCN29012.1	144.49	19	0	0	17	ND	ND	1.67E+04	11	0	0	17	67259
rod shape-determining protein MreB [*Klebsiella pneumoniae* subsp. *pneumoniae*]	KPV70631.1	128.89	29	4	3	20	0	0	9.76E+03	10	1	1	9	36952
cell division protein FtsY [*Klebsiella pneumoniae* subsp. *pneumoniae*]	AIX81397.1	76.83	7	0	0	7	ND	ND	1.24E+03	4	0	0	6	54942
cell division protein FtsN [*Klebsiella pneumoniae* subsp. *pneumoniae*]	AIX76771.1	76.1	13	0	0	13	ND	ND	2.15E+03	2	0	0	2	31904
cell division protein FtsL [*Klebsiella pneumoniae* subsp. *pneumoniae*]	AIK81082.1	75.78	45	0	0	45	ND	ND	1.08E+03	2	0	0	4	8068
cell division protein MukB [*Klebsiella pneumoniae* subsp. *pneumoniae*]	AMA15804.1	72.69	4	0	0	2	ND	ND	9.50E+02	6	0	0	5	169528
Cell division protein FtsA [*Klebsiella pneumoniae* subsp. *pneumoniae*]	AKE77598.1	64.03	10	0	0	2	ND	ND	3.05E+02	4	0	0	1	45314

cell division protein FtsP [*Klebsiella pneumoniae* subsp. *pneumoniae*]	OCO39682.1	53.99	6	0	0	3			9.85E+02	2	0	0	2	51928
**Cell wall and membrane**														
outer membrane lipocarrier protein LolA [*Klebsiella pneumoniae* subsp. *pneumoniae*]	AIK80182.1	148.87	53	0	0	51	ND	ND	5.53E+04	9	0	0	12	21227
lipoprotein [*Klebsiella pneumoniae* subsp. *pneumoniae*]	OCN93024.1	129.78	49	5	0	44	3.14E+02	ND	2.50E+04	8	1	0	13	18579
peptidoglycan-associated outer membrane lipoprotein [*Klebsiella pneumoniae* subsp. *pneumoniae*]	AMV57997.1	101.59	29	0	0	29	ND	ND	2.54E+04	4	0	0	8	18863
murein L D-transpeptidase [*Klebsiella pneumoniae* subsp. *pneumoniae*]	AIX79658.1	152.25	32	6	3	28	1.51E+03	4.22E+03	1.58E+04	12	2	2	18	35353
L D-transpeptidase [*Klebsiella pneumoniae* subsp. *pneumoniae*]	OCO04225.1	95.03	22	3	3	16	4.53E+02	2.56E+03	1.17E+04	7	2	2	8	32928
exported protein required for envelope biosynthesis and integrity [*Klebsiella pneumoniae* subsp. *pneumoniae*]	AIK81120.1	152.25	26	0	0	22	ND	ND	1.03E+04	16	0	0	17	84014
serine-type D-Ala-D-Ala carboxypeptidase [*Klebsiella pneumoniae* subsp. *pneumoniae*]	OCN40167.1	149.41	31	2	2	25	ND	ND	9.28E+03	11	1	1	13	43927
murein transglycosylase [*Klebsiella pneumoniae* subsp. *pneumoniae*]	OCO07900.1	133.41	26	0	0	26	ND	ND	1.01E+04	14	0	0	15	73160
glucosyltransferase MdoH [*Klebsiella pneumoniae* subsp. *pneumoniae*]	AMV52513.1	116.97	10	0	0	10	ND	ND	4.33E+03	8	0	0	9	96048
UTP--glucose-1-phosphate uridylyltransferase [*Klebsiella pneumoniae* subsp. *pneumoniae*]	AHJ80480.1	116.96	26	0	0	18	ND	ND	7.16E+03	7	0	0	6	33086
UTP--glucose-1-phosphate uridylyltransferase [*Klebsiella pneumoniae* subsp. *pneumoniae*]	AHJ80480.1	116.96	26	0	0	18	ND	ND	7.16E+03	7	0	0	6	33086
membrane biogenesis protein [*Klebsiella pneumoniae* subsp. *pneumoniae*]	OCN26347.1	81.47	22	0	0	15	ND	ND	1.21E+03	5	0	0	3	27884
3-deoxy-8-phosphooctulonate synthase [*Klebsiella pneumoniae* subsp. *pneumoniae*]	OCO39254.1	97.09	25	0	0	19	ND	ND	9.42E+03	7	0	0	7	30825
inner-membrane protein insertion factor [*Klebsiella pneumoniae* subsp. *pneumoniae*]	AIK81915.1	79.28	7	0	0	5	ND	ND	5.71E+03	3	0	0	4	59502
L D-transpeptidase [*Klebsiella pneumoniae* subsp. *pneumoniae*]	AMV59587.1	112.26	29	0	5	20	ND	0	3.99E+03	9	0	2	6	33952
outer membrane protein assembly factor BamA [*Klebsiella pneumoniae* subsp. *pneumoniae*]	OCO00265.1	147.23	22	0	0	18	ND	ND	3.56E+03	15	0	0	16	90056
rare lipoprotein LptE [*Klebsiella pneumoniae* subsp. *pneumoniae*]	AIK80465.1	122.01	53	20	0	53	0	ND	2.30E+03	5	1	0	5	17953
membrane protein [*Klebsiella pneumoniae* subsp. *pneumoniae*]	AIX84745.1	68.5	15	0	0	9	ND	ND	1.69E+03	2	0	0	3	18126
UDP-N-acetylglucosamine--N-acetylmuramyl-(pentapeptide) pyrophosphoryl-undecaprenol N-acetylglucosamine transferase [*Klebsiella pneumoniae* subsp. *pneumoniae*]	KHF64275.1	56.83	7	0	0	3	ND	ND	1.63E+03	2	0	0	1	37114
Lipopolysaccharide biosynthesis protein WzzE [*Klebsiella pneumoniae* subsp. *pneumoniae*]	AKE78256.1	79.53	11	0	0	11	ND	ND	2.39E+03	3	0	0	4	38239
UDP-N-acetylmuramoylalanyl-D-glutamate--2 6-diaminopimelate ligase [*Klebsiella pneumoniae* subsp. *pneumoniae*]	AIX77478.1	85.76	12	2	2	5	3.84E+02	5.87E+02	1.94E+03	5	1	2	2	53329
OmpA family lipoprotein [*Klebsiella pneumoniae* subsp. *pneumoniae*]	OCN80760.1	101.59	23	0	0	23	ND	ND	3.21E+03	3	0	0	6	22384
outer membrane receptor protein [*Klebsiella pneumoniae* subsp. *pneumoniae*]	OAK87561.1	68.52	6	0	0	6	ND	ND	1.44E+03	3	0	0	3	82325

gram-negative pili assembly chaperone C-terminal domain protein [*Klebsiella pneumoniae* subsp. *pneumoniae*]	AIK82776.1	60.07	11	0	0	11	ND	ND	1.33E+03	2	0	0	2	23976
Outer membrane protein assembly factor BamA precursor [*Klebsiella pneumoniae* subsp. *pneumoniae*]	KHF66979.1	143.07	21	0	0	18	ND	ND	7.38E+02	15	0	0	16	89983
inner membrane protein YhjD [*Klebsiella pneumoniae* subsp. *pneumoniae*]	OCN96551.1	49.87	10	0	0	4	ND	ND	1.58E+03	2	0	0	1	38541
lipopolysaccharide ABC transporter permease [*Klebsiella pneumoniae* subsp. *pneumoniae*]	AIW69132.1	68.04	7	0	0	7	ND	ND	1.47E+03	2	0	0	2	39630
UDP-N-acetylglucosamine 1-carboxyvinyltransferase [*Klebsiella pneumoniae* subsp. *pneumoniae*]	KLA37987.1	37.67	5	0	0	5	ND	ND	1.93E+03	2	0	0	2	44585
beta-hexosaminidase [*Klebsiella pneumoniae* subsp. *pneumoniae*]	AIK79984.1	71.5	11	0	0	7	ND	ND	7.76E+02	3	0	0	3	36972
membrane biogenesis protein [*Klebsiella pneumoniae* subsp. *pneumoniae*]	KPV70227.1	68.64	11	0	0	9	ND	ND	1.55E+03	3	0	0	2	42056
Outer-membrane lipoprotein LolB precursor [*Klebsiella pneumoniae* subsp. *pneumoniae*]	KHF51907.1	58.14	7	0	0	7	ND	ND	5.26E+02	1	0	0	1	23032
N-acetylmuramoyl-L-alanine amidase [*Klebsiella pneumoniae* subsp. *pneumoniae*]	AMV59108.1	50.39	6	0	0	3	ND	ND	5.66E+02	2	0	0	1	48350
UDP-4-amino-4-deoxy-L-arabinose--oxoglutarate aminotransferase [*Klebsiella pneumoniae* subsp. *pneumoniae*]	OCO29265.1	41.21	3	0	0	0	ND	ND	ND	1	0	0	0	41246
UDP-N-acetylmuramate--alanine ligase [*Klebsiella pneumoniae* subsp. *pneumoniae*]	AIK81073.1	37.68	5	0	0	2	ND	ND	1.41E+02	2	0	0	1	51765
alpha-D-glucose-1-phosphatase [*Klebsiella pneumoniae* subsp. *pneumoniae*]	AMV59457.1	55.41	10	0	0	10	ND	ND	6.28E+02	2	0	0	2	22705
periplasmic binding and sugar binding domain of LacI family protein [*Klebsiella pneumoniae* subsp. *pneumoniae*]	AIK79103.1	57.84	7	0	0	7	ND	ND	2.55E+02	2	0	0	3	36304
Periplasmic murein peptide-binding protein precursor [*Klebsiella pneumoniae* subsp. *pneumoniae*]	AKE76347.1	80.23	11	0	0	8	ND	ND	5.14E+02	4	0	0	3	59646
